# Museum Genomics Illuminate the High Specificity of a Bioluminescent Symbiosis for a Genus of Reef Fish

**DOI:** 10.3389/fevo.2021.630207

**Published:** 2021-02-04

**Authors:** Alison L. Gould, Allison Fritts-Penniman, Ana Gaisiner

**Affiliations:** California Academy of Sciences, San Francisco, CA, United States

**Keywords:** symbiosis, bioluminescence, coral reef fish, photobacterium, *Siphamia tubifer*, symbiont specificity, co-diversification, museum genomics

## Abstract

Symbiotic relationships between bioluminescent bacteria and fishes have evolved multiple times across hundreds of fish taxa, but relatively little is known about the specificity of these associations and how stable they are over host generations. This study describes the degree of specificity of a bioluminescent symbiosis between cardinalfishes in the genus *Siphamia* and luminous bacteria in the Vibrio family. Primarily using museum specimens, we investigated the codivergence of host and symbiont and test for patterns of divergence that correlate with both biogeography and time. Contrary to expectations, we determined that the light organ symbionts of all 14 *Siphamia* species examined belong to one genetic clade of *Photobacterium mandapamensis* (Clade II), indicating that the association is highly specific and conserved throughout the host genus. Thus, we did not find evidence of codivergence among hosts and symbionts. We did observe that symbionts hosted by individuals sampled from colder water regions were more divergent, containing more than three times as many single nucleotide polymorphisms than the rest of the symbionts examined. Overall, our findings indicate that the symbiosis between *Siphamia* fishes and *P. mandapamensis* Clade II has been highly conserved across host taxa and over a broad geographic range despite the facultative nature of the bacterial symbiont. We also present a new approach to simultaneously recover genetic information from a bacterial symbiont and its vertebrate host from formalin-fixed specimens, enhancing the utility of museum collections.

## INTRODUCTION

Bioluminescent symbioses have evolved multiple times across diverse fish and squid taxa, including at least 17 times in the ray-finned fishes (Dunlap and Urbanczyk, 2013; Davis et al., 2016). Approximately 500 species of fish are known to be symbiotically bioluminescent, but the range of luminous bacteria they associate with is much narrower than what would be predicted based solely on the diversity and relative abundances of bacteria in the surrounding seawater. For example, both leiognathid fishes and anglerfishes representing four different families have each been described to host only two species of closely related luminous bacteria (Reichelt et al., 1977; Kaeding et al., 2007; Baker et al., 2019). A similar level of bacterial specificity has been described in at least seven additional fish families with symbiotically luminous members (Dunlap et al., 2007). Despite this seemingly high level of specificity for environmentally transmitted symbioses, our understanding of these associations is lacking, as the majority have yet to be characterized. Moreover, luminous bacterial symbionts are generally not obligately dependent on their host (but see Hendry et al., 2014, 2018) and can survive in a variety of other habitats including seawater, sediment, and the surfaces and digestive tracts of various marine organisms. Thus, these facultative symbionts must retain the genetic machinery necessary to associate with their hosts while also being able to compete with the rest of the microbial community in the environment (Bright and Bulgheresi, 2010), bringing up questions of the evolutionary history of these associations and the mechanisms that maintain specificity.

Luminous bacteria involved in bioluminescent symbioses are members of the *Vibrionaceae* family, and of these, three species of *Photobacterium* are known to associate with fish hosts: *P. kishitanii, P. leiognathi*, and *P. mandapamensis. Photobacterium leiognathi* and *P. mandapamensis* are closely related and phenotypically similar and were once considered to be the same species based on the inability of 16S rRNA and *gyrB* genes to distinguish between them (Ast and Dunlap, 2004). Ecologically, however, symbiotic strains of *P. leiognathi* and *P. mandapamensis* are distinct; *P. mandapamensis* has a broad host range and has been identified from the light organs of fishes from at least four families representing two teleost orders collected from diverse marine habitats and depths, while *P. leiognathi* primarily associates with various leiognathid fishes (Kaeding et al., 2007). Furthermore, an analysis of the *lux* genes, which are responsible for light production, indicated that *luxF* is present in *P. mandapamensis* yet absent in *P. leiognathi*. Thus, these two groups were considered to be distinct subspecies of *P. leiognathi* (Ast and Dunlap, 2004). An additional analysis of various strains of *P. mandapamensis* and *P. leiognathi* isolated from fish light organs further discriminated *P. mandapamensis* from *P. leiognathi* and revealed two distinct clades within *P. mandapamensis* (Clades I and II) based on *gyrB* and *luxAB(F)E* genes (Kaeding et al., 2007), although the physiological and ecological significance of these two clades remain unknown. In that study, a high degree of specificity was also observed between the the cardinalfish host *Siphamia tubifer* and Clade II of *P. mandapamensis*.

Bioluminescence has evolved multiple times within the cardinalfish family (Perciformes: Apogonidae), but only the genus *Siphamia* rely on a symbiotic relationship with bacteria to produce light; other cardinalfishes produce their own light presumably via the acquisition of luciferin from their diet (Thacker and Roje, 2009). *Siphamia* consists of 25 species all of which are symbiotically bioluminescent (Thacker and Roje, 2009; Gon and Allen, 2012). *Siphamia* fishes possess a ventral light organ connected to the intestine, which contains a dense population of luminous bacteria (~10^8^ cells) (Dunlap and Nakamura, 2011). The light organ symbionts of only one *Siphamia* species, *S. tubifer*, originating from a small region in the Okinawa Islands, Japan, have been characterized to date; of the hundreds of specimens examined, all hosted members of Clade II of *P. mandapamensis* in their light organs, suggesting a high level of specificity for this association (Kaeding et al., 2007; Gould and Dunlap, 2019). However, *S. tubifer* is broadly distributed throughout the Indo-Pacific, spanning from eastern Africa to the French Polynesian Islands (Gon and Allen, 2012), thus the degree of specificity of the association across its broad geographic range remains unknown. Furthermore, the luminous symbionts of the other 24 *Siphamia* species have yet to be identified.

**FIGURE 1 |:**
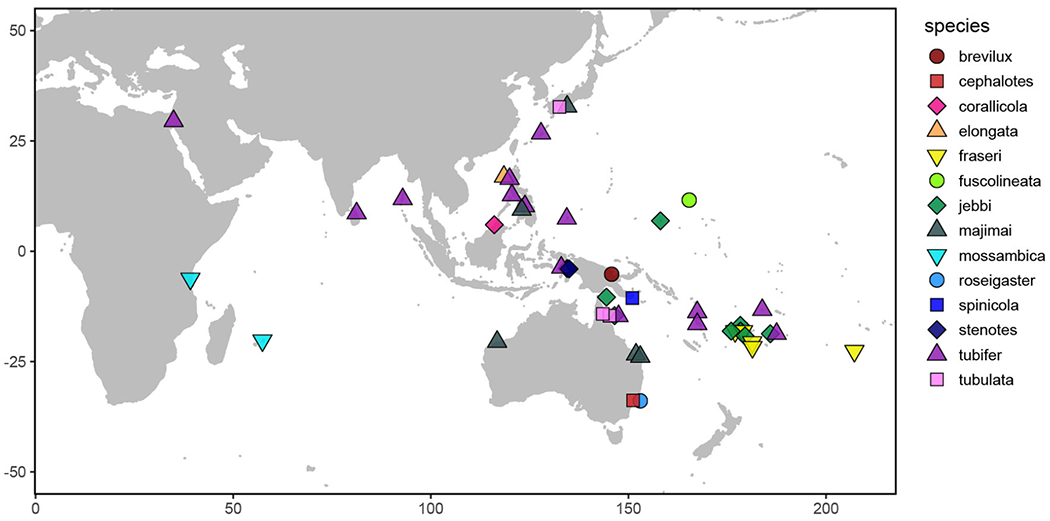
Map depicting the sampling locations of the *Siphamia* specimens examined in this study. Distinct colors and shapes represent the different *Siphamia* species sampled as indicated in the figure legend.

The primary goals of this study were to characterize the degree of specificity of the bioluminescent symbiosis throughout the *Siphamia* genus and across the broad geographic range of *S. tubifer*. More specifically, we wanted to determine whether the high degree of specificity observed for *S. tubifer* and members of Clade II of *Photobacterium mandpamensis* (Kaeding et al., 2007) is maintained across the host genus, and if not, whether any observed symbiont diversity is a result of codivergence between host and symbiont. By leveraging readily available museum specimens from several natural history collections we were able to sample geographically and temporally diverse *Siphamia* taxa to address these questions (Figure 1). However, recovering genetic information from specimens in wet collections, particularly those initially fixed in formalin, is challenging primarily due to DNA degradation and cross-linkage. Here we present a new approach to simultaneously recover genetic information from a bacterial symbiont and its vertebrate host from formalin-fixed specimens based on a combination of recently developed methods. This allowed us to sample diverse *Siphamia* taxa spanning decades throughout the Indo-Pacific as well as *S. tubifer* from locations throughout its entire geographic range (Figure 1). With these samples we were able to test for patterns of symbiont diversity at the subspecies level that correlate with host biogeography, temperature and time. Although codivergence is unlikely to occur when the symbiont has a free-living stage (Ronquist, 1998) and has not been documented for other symbiotically bioluminescent fishes (Dunlap et al., 2007), our ability to recover informative genetic information from both the host and symbiont also allowed us to test for evidence of codivergence in this bioluminescent association.

## METHODS

### Taxon Sampling and DNA Extraction

**FIGURE 2 |:**
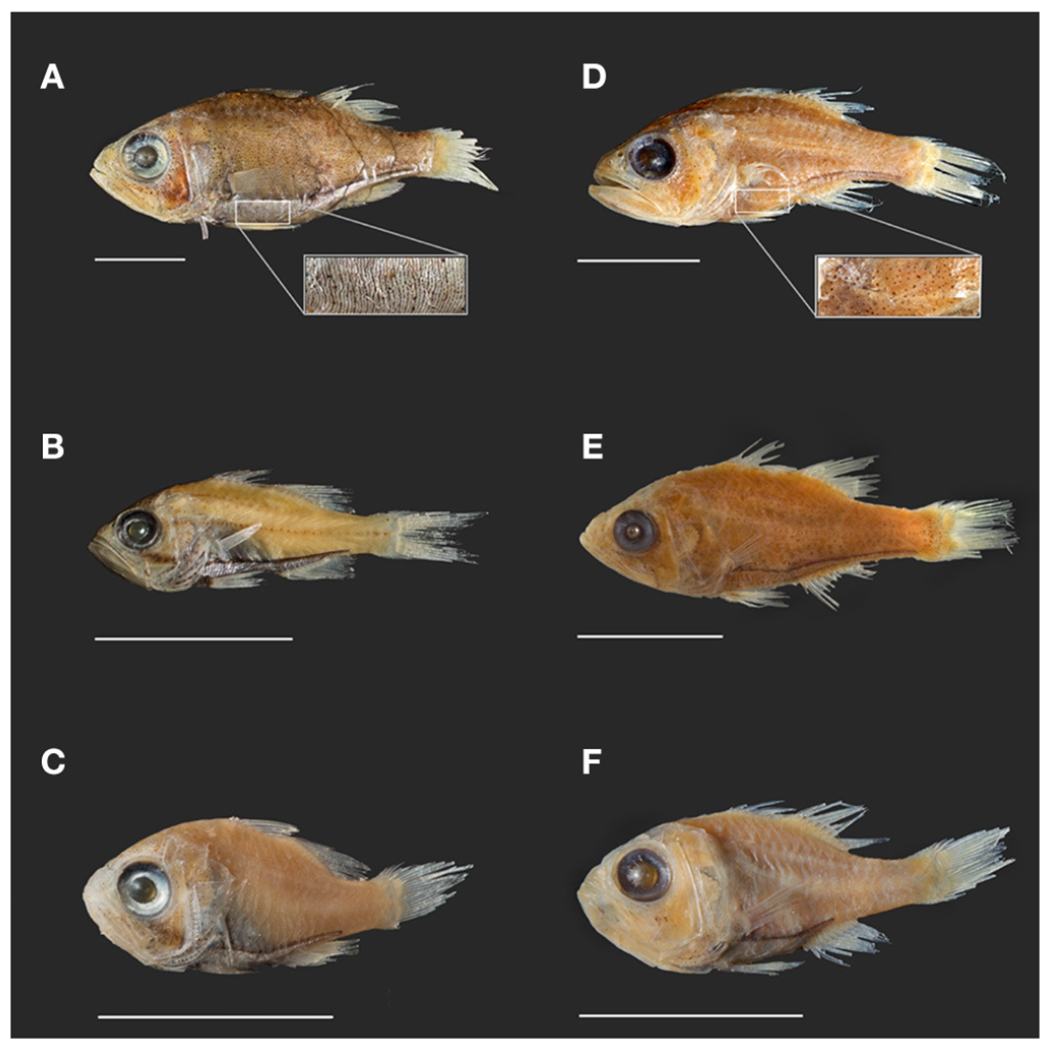
Photographs of select *Siphamia* specimens from lots used in this study. Specimens **(A–C)** represent the *tubifer* subgroup (Gon and Allen, 2012), identified by the striated light organ **(A)** and specimens **(D–F)** represent the *tubulata* subgroup, identified by the spotted light organ **(D)**. **(A)** *S. tubifer* (USNM341595) with insert of light organ detail showing striated morphology. **(B)** *S. stenotes* (USNM396981, paratype). **(C)** *S. jebbi* (CAS223855). **(D)** *S. tubulata* (CAS28515) with insert of light organ detail showing spotted morphology. **(E)** *S. corallicola* (USNM203781). **(F)** *S. brevilux* (CAS65338, paratype). Scale bars indicate 1 cm in length.

We sampled 59 specimens representing 14 *Siphamia* species obtained from the combined wet collections of the California Academy of Sciences, the Australian Museum, and the Smithsonian National Museum of Natural History, and including several newly collected specimens deposited at the California Academy of Sciences (Figures 1, 2, Table 1). The specimens collected for this study were stored in 96% ethanol and were obtained with appropriate collection permits, following approved protocols and permits for the capture, care and handling of fish by the California Academy of Science’s Institutional Animal Care and Use Committee. To extract DNA from each specimen, we adapted the following protocol from two previous methods designed for recovering DAN from formalin-fixed tissues (Hykin et al., 2015; Ruane and Austin, 2017). Light organs were aseptically dissected and individually placed into 1 ml of GTE buffer and allowed to soak for 3 h at room temperature. This step was repeated two times, after which each light organ was transferred into a final 1 ml aliquot of fresh GTE buffer and left to soak overnight at room temperature. The following morning, each sample was transferred into 1 ml of 100% ethanol for 1 min, followed by 1 ml of 70% ethanol for 5 min, and 1 ml of nuclease-free water for 10 min at room temperature. Light organs were then transferred into 180 ul of pre-heated (98°C) ATL buffer (QIAGEN) and incubated at 98°C for 15 min, after which samples were immediately placed on ice for at least 2 min. Once cooled, 40 ul of proteinase K was added to each sample, and the samples were incubated at 60°C for 48 h on a shaking heat block. Samples were vortexed periodically and additional 20 ul aliquots of proteinase K were added as needed (up to 100 ul total). Following this incubation period, DNA was extracted using the QIAGEN DNEasy Blood and Tissue Kit as described by the manufacturer. Purified DNA products were eluted into 50 ul of nuclease-free water after a 3-min incubation at 55°C.

### Library Preparation and Sequencing

Samples were quantified using the Qubit dsDNA HS Assay Kit on the Qubit 2.0 Fluorometer (Invitrogen) and profiled with an Agilent 2100 Bioanalyzer. Samples with a peak in size distribution >300 bp were sonicated with a Qsonica (Q800R3) for one or two minutes (if peak was >1,500 bp) with a pulse rate of 10–10 s and an amplitude of 25%. Samples were then treated with the NEBNext^©^ FFPE DNA Repair Mix following the manufacturer’s instructions and DNA libraries were immediately prepped using the NEBNext^©^ Ultra II DNA Library Prep Kit. Samples with low or undetectable quantities of dsDNA were re-quantified using the Qubit ssDNA HS Assay Kit and prepared using the Accel-NGS 1S Plus DNA Library Kit (Swift Biosciences), which uses both single- and double-stranded DNA as templates. Each sample was uniquely indexed with the NEBNext^©^ Multiplex Oligos for Illumina. Final libraries were cleaned with AMPure XP magnetic beads, pooled, and sequenced as single-end 150 bp (UC Berkeley, QB3) or paired-end 150 bp reads on the Illumina HiSeq 4000 platform (NovoGene), or as paired-end 150 bp reads on the Illumina NovaSeq S4 platform (Genewiz). Supplementary Table 1 contains details for each sample and library preparation.

### Sequence Analysis

Sequences were demultiplexed, trimmed and quality filtered for a Phred score of 20 or above using Trimmomatic (Bolger et al., 2014). The remaining reads were aligned to the reference genome of *Photobacterium mandapamensis*, isolated from the light organ of *Siphamia tubifer* (Urbanczyk et al., 2011) with BWA-MEM using the default parameters (Li, 2013). Unaligned sequences were then processed with MitoFinder (Allio et al., 2020) using the reference mitochondrial genome of the Banggai cardinalfish *Pterapogon kauderni* (Matias and Hereward, 2018). All cardinalfish cytochrome oxidase subunit 1 (*COI*) gene sequences that were recovered were aligned using MUSCLE (Edgar, 2004) and a maximum likelihood analysis was carried out with raxml-ng (Kozlov et al., 2019) using the evolutionary model TIM2+F+I+G4, which had the lowest BIC score as predicted by IQtree (Nguyen et al., 2015), and 1,000 bootstraps to infer the phylogenetic relationships between host species. *COI* sequences of *Siphamia* spp. from previous studies were also included in the analysis (Supplementary Table 2). An additional phylogeny was inferred from a supermatrix of 15 mitochondrial genes (*ATP6, ATP8, COXI, COX2, COX3, CYTB, ND1, ND2, ND3, ND4, ND4L, ND5, ND6, 16S, 18S*) identified by MitoFinder that were present in at least 70% of the individuals included in the analysis using the SuperCRUNCH python toolkit (Portik and Wiens, 2020). The concatenated supermatrix alignment was used in a maximum likelihood analysis by raxml-ng with 500 bootstrap replicates and the evolutionary model TIM2+F+R4 as predicted by IQtree to infer the phylogenetic relationships between species.

For the aligned symbiont reads, two approaches were used to determine symbiont identity based on consensus sequences generated for each sample using the samtools mpileup command and the bcftools consensus caller (Li et al., 2009). First, the program fastANI (Jain et al., 2018) was used to calculate the whole-genome average nucleotide identity (ANI) of each sample relative to several available reference genomes of *Photobacterium* species previously isolated from fish light organs, *P. kishitanii* pjapo1.1, *P. leiognathi* lrivu4.1, *P. mandapamensis* ajapo4.1 (Clade I), *P. mandapamensis* gjord1.1 (Clade II), and *P. mandapamensis* svers1.1 (Clade II). The output represents the percent ANI of all orthologous genes shared between each sample’s consensus genome and the reference genomes. We also extracted 16S rRNA gene sequences from each sample by aligning all filtered light organ sequences to the complete 16S sequence of a free-living strain of *Photobacterium leiognathi* (AY292917) (Nishiguchi and Nair, 2003). We chose this strain of *P. leiognathi* to capture a range of potential luminous bacteria that could be present in the light organ. A sequence similarity search was then performed with the basic local alignment search tool (BLAST) (Altschul et al., 1990) against NCBI’s microbial database to identify the known sequence with the lowest E-value and highest percent identity.

To infer the phylogenetic relationships between symbionts from different hosts, all sequences that aligned to the reference genome of *P. mandapamensis* (Urbanczyk et al., 2011) were also analyzed for sequence variation with the program snippy (Seemann, 2015). Locus filters required a minimum depth of 10x and presence in at least 90% of all reads to minimize calling errors due to the degraded nature of many museum samples. In snippy, single nucleotide polymorphisms (SNPs) were called by Freebayes, and consensus SNP haplotypes were generated for each sample, presumably representing the most abundant symbiont. A sequence alignment of a core set of these SNPs was then created across all samples, filtering for those that had enough genome coverage to produce a core set of at least 1,000 SNPs. SNPs were also called for two additional reference genomes of *P. mandapamensis* representing both Clade I (ajapo4.1) and Clade II (Res4.1) and were included in the core set of SNPs as phylogenetic references. The phylogenetic relationships of these bacteria were then inferred with raxml-ng (Kozlov et al., 2019) using the evolutionary model TVM+F+R3, which had the lowest *BIC* score as predicted by IQtree (Nguyen et al., 2015), and 1,500 bootstrap replicates.

Samples included in both the host and symbiont phylogenies were then compared and tested for co-divergence using the cospeciation function in the R phytools package (Revell, 2011). SNPs were annotated with the program SNPeff (Cingolani et al., 2012). Pairwise phylogenetic (patristic) distances between symbionts were calculated with the adephylo package (Jombart et al., 2010) in R, and pairwise geographic distances were calculated based on each specimen’s latitude and longitude using the R package geodist (Padgham and Sumner, 2020). Tests for correlations between the phylogenetic distances for each pair of symbionts and their geographic distance or difference between sampling years were carried out, and P-values were adjusted for multiple comparisons with the Holm method in R.

## RESULTS

### DNA Recovery

Variable amounts of total DNA were recovered from the light organs of preserved *Siphamia* specimens, ranging from undetectable levels (<2 ng) to more than 1,500 ng. Although light organ size (~1–3 mm diameter) increases linearly with fish standard length (Gould et al., 2016), there was no correlation between DNA yield and specimen size (Spearman’s rank correlation: rho=0.52, *P* = 0.09). Despite this variability in yield, quality DNA sequences were recovered from several specimens with undetectable levels of starting DNA. In fact, some samples with undetectable levels of input DNA resulted in >90% coverage of the symbiont genome at 10x depth. Of note, many of those sequence libraries were prepared using the Swift Bioscience Accel-NGS 1S Plus DNA Library Kit which uses both double and single stranded DNA as the starting template (Supplementary Table 1).

### Host Phylogeny

**FIGURE 3 |:**
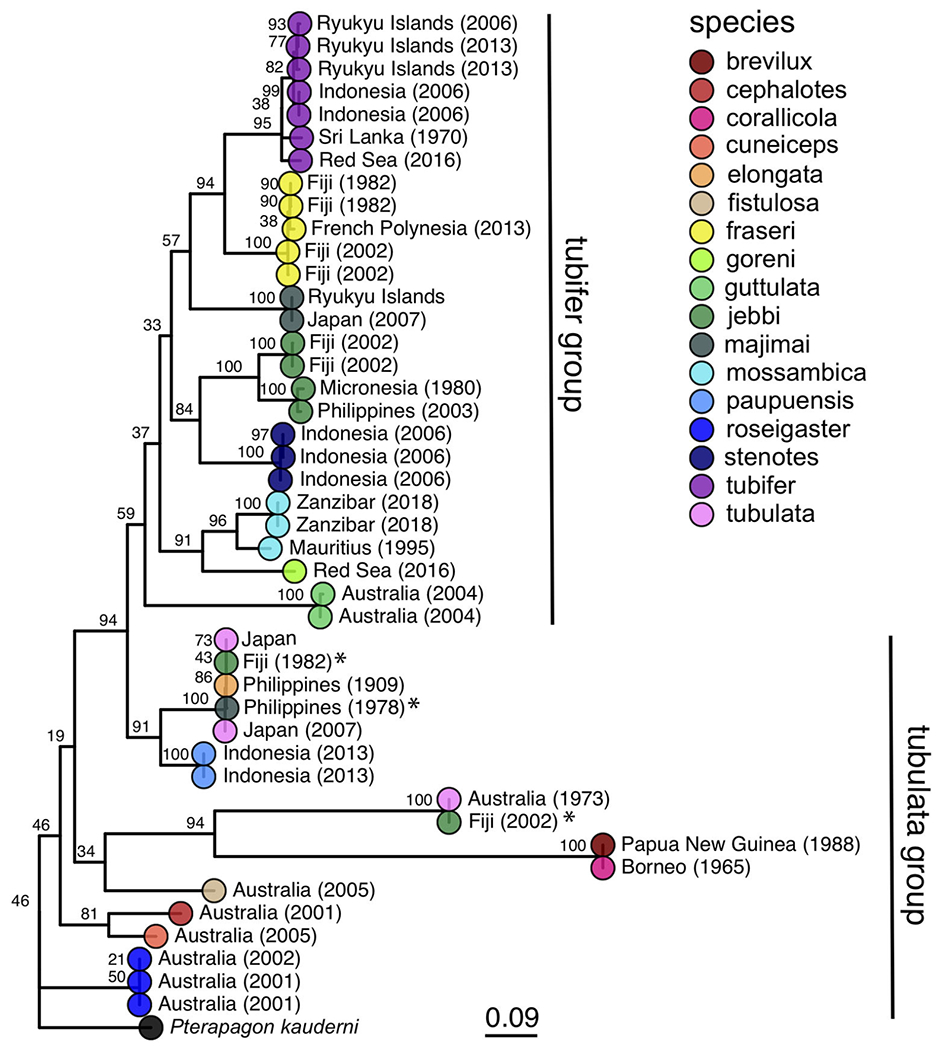
Maximum likelihood phylogeny of cardinalfishes in the genus *Siphamia* based on *COI* gene sequences. Species identities are indicated by the branch tip colors and the sampling location and year of each specimen is listed in the branch label. Bootstrap support values are indicated at each node. The Banggai cardinalfish, *Pterapogon kauderni*, was used as the outgroup. The *tubifer* and *tubulata* subgroups within *Siphamia* (Gon and Allen, 2012) are highlighted with vertical lines to the right of the tree. Specimens that fall outside of their designated subgroup based on species identities are indicated with an *.

Host *COI* sequences were recovered from 32 samples and analyzed with an additional 12 *Siphamia COI* sequences from previous studies (Supplementary Table 2) to generate a maximum likelihood phylogeny of 17 *Siphamia* species (Figure 3). The supermatrix of 15 mitochondrial genes from 27 *Siphamia* specimens representing 11 species resulted in a phylogenetic tree with similar, but not identical, topology, and stronger bootstrap support at the nodes (Supplementary Figure 1).

Our phylogenetic hypothesis for *Siphamia* is very similar to that proposed by Gon and Allen (2012) using morphological characters, with slight variations in the placement of specific taxa. Our tree contains a clade that corresponds to Gon and Allen’s *S. tubifer* species group, characterized by a striated pattern on the light organ (Figure 2), although one individual *S. majimai* and two *S. jebbi* specimens fell out of this group (Figure 3). Within this group, our trees support the relationships of *S. jebbi* and *S. stenotes* as sister species, as well as *S. tubifer* and *S. fraseri*. The relative placement of *S. mossambica, S. majimai*, and *S. goreni* varies among the trees, but there is support for *S. mossambica* and *S. goreni* as sister species in the *COI* tree, *S. mossambica* and *S. majimai* as sisters in the supermatrix tree, and *S. majimai* and *S. goreni* as sisters in the morphological tree (Gon and Allen, 2012). As such, it is likely that all of three of these species belong to one clade. The relationships among the species outside of the *S. tubifer* group are less certain, with several species clustering into species complexes. However, *S. roseigaster, S. cuneiceps*, and *S. cephalotes* consistently fall out near the base of the tree, indicating that these species diverged earlier.

### Symbiont Identification and Phylogeny

To identify the most abundant light organ symbiont of each *Siphamia* host, we first calculated the ANI of each sample’s consensus symbiont genome relative to the reference genomes of several symbiotic strains of *Photobacterium* sp. isolated from fish light organs. Eighty-six percent of all the symbionts in this study had ANI values of 95% or greater relative to *P. mandapamensis* strains in Clade II (gjord1.1 and svers1.1) (Table 2), which is the recommended value to delimit bacterial species (Goris et al., 2007). All remaining samples also had highest ANI values relative to *P. mandapamensis* Clade II than to the other *Photobacterium* reference strains, with the exception of one sample (AMI18740-066), which was most similar to *P. mandapamensis* Clade I (86.8% ANI vs. 85.4% ANI to Clade II) but had fewer orthologous matches (41.7% to Clade I vs. 62.3% to Clade II). None of the symbionts had higher ANI values relative to *P. leiognathi* than to *P. mandapamensis* strains. However, many samples had low overall genome coverage, and thus, the ANI calculations are based on only small numbers of orthologous regions (Table 2). To confirm the identities of the light organ symbionts, we also recovered 16S rRNA sequences from the shotgun sequence data and matched them against the NCBI database. Ninety three percent of all samples had >95% coverage of the 16S rRNA gene at 10x read depth. Of these, 67% returned *P. mandapamensis* as their top hit, and all others returned *P. leiognathi* (Supplementary Table 3). Importantly, 16S rRNA gene sequences alone are insufficient at discriminating between strains of *P. mandapamensis* and *P. leiognathi* (Ast and Dunlap, 2004; Wada et al., 2006). No other *Photobacterium* species were identified as top hits in this analysis.

Single nucleotide polymorphisms (SNPs) were detected for the light organ symbionts from most of the specimens sampled, but this number varied greatly and correlated with the variability in genome coverage (Spearman’s rank correlation: rho = 0.84, *P* < 0.001, Supplementary Table 1). Samples with >50% symbiont genome coverage at 10x read depth had an average of 23,221 SNPs relative to the reference genome of *P. mandapamensis* (Urbanczyk et al., 2011). A core set of 1,471 SNPs were identified across 32 specimens that represent 11 *Siphamia* host species and included reference genomes from both Clade I and Clade II of *Photobacterium mandapamensis*. Sixty-eight percent of these SNPs were synonymous, and the remaining non-synonymous SNPs were found in 288 distinct genes. None of the core SNPs were located in the *lux* operon, composed of the genes responsible for light production. However, two non-synonymous SNPs were detected in the *rpoN* gene, which is known to play a role in biofilm formation, bioluminescence, and symbiosis initiation for *Aliivibrio fischeri* (Wolfe et al., 2004), the luminous symbiont of many squid and other fish species. No other SNPs were detected in genes of known function for the bioluminescent symbiosis between *A. fischeri* and the squid host *Euprymna scolopes* (Norsworthy and Visick, 2013).

**FIGURE 4 |:**
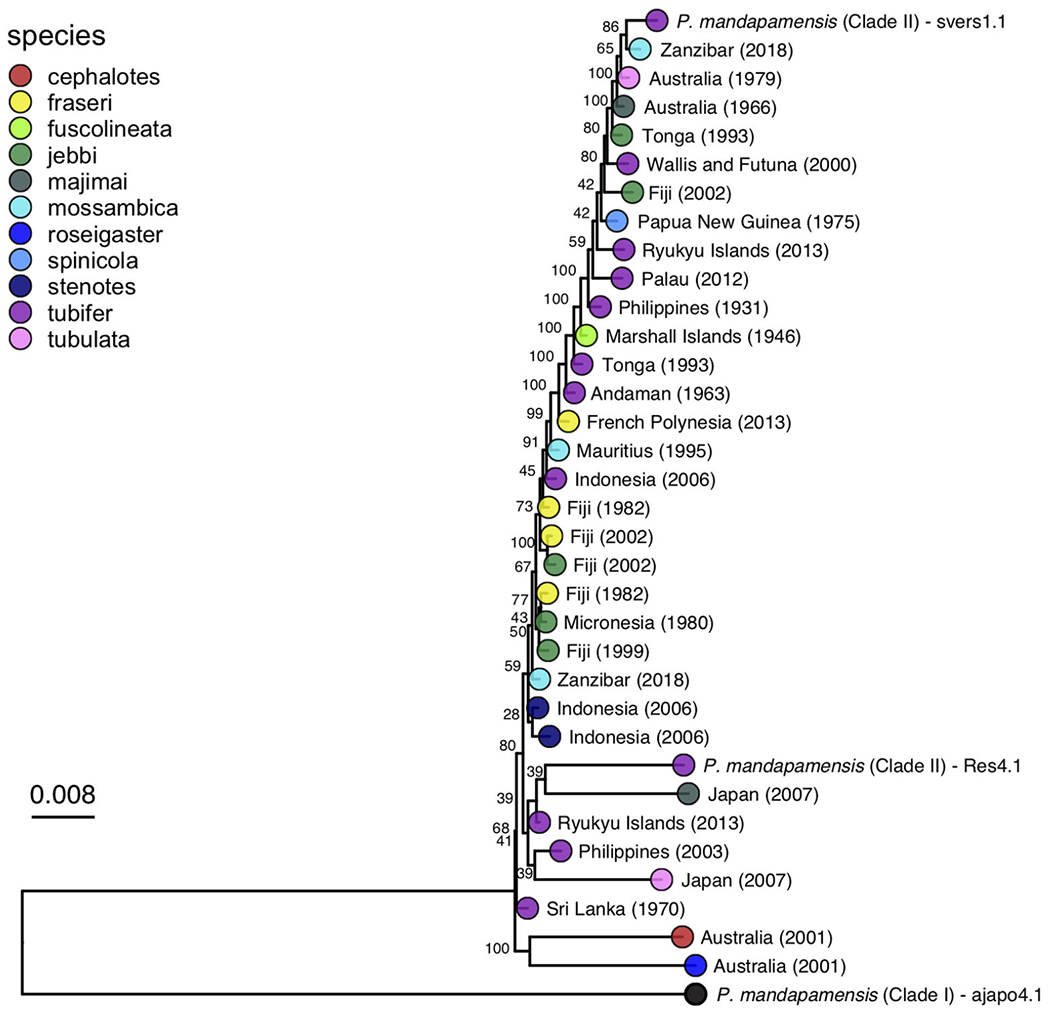
Maximum likelihood phylogeny of the light organ symbionts of various *Siphamia* species constructed from a core set of 1,471 single nucleotide polymorphisms. Corresponding host species are indicated by the branch tip colors and the sampling location and year of each specimen is listed in the branch label. Bootstrap support values are indicated at each node.

A maximum likelihood phylogeny was inferred for the bacterial symbionts using full sequence alignments that included the core set of SNPs described above. This analysis confirmed that all *Siphamia* light organ symbionts for which we recovered enough quality sequence data to analyze belong to Clade II of *P. mandapamensis* and that the reference strain of *P. mandapamensis* representing Clade I (ajapo4.1) was a clear outgroup (Figure 4). The majority of symbionts analyzed were closely related to the reference strain svers1.1 of *P. mandapamensis*, although several symbionts fell out in a group with *P. mandapamensis* strain Res 4.1, both of which are members of Clade II. There were three additional symbionts, all from different host species, that did not belong to either of these subgroups, but are still clearly members of Clade II. We carried out an additional analysis that also included four individuals initially excluded from the analysis because they produced a smaller set of core SNPs (*N* = 166). The topology of the resulting phylogeny did not change, and these additional symbionts also belong to Clade II of *P. mandapamensis* (Supplementary Figure 2).

No clear patterns of symbiont divergence that corresponded with host species, geography, or time emerged. There was no correlation between symbiont phylogenetic distance and geographic distance (Spearman’s rank correlation: rho=−0.013, P_corr_ =1) and there was a slightly negative correlation between phylogenetic distance and time in years (Spearman’s rank correlation: rho = −0.17, P_corr_ =0.006). In fact, the oldest specimen for which informative sequence data was retained was collected in 1931 and it had luminous bacteria in its light organ that was highly similar to symbionts from specimens collected more than 80 years later. Similarly, *Siphamia* specimens collected from locations in the western Indian Ocean had symbionts that were closely related to those from locations as far east as Fiji and French Polynesia. With respect to *S. tubifer*, which has the broadest geographic distribution of all *Siphamia* species, the symbionts of all ten specimens included in the symbiont phylogeny fell out in Clade II of *P. mandapamensis* and showed no pattern of strain diversity by geography, confirming the high degree of specificity of this association, even across *S. tubifer’s* broad geographic range.

The bacterial symbionts from four distinct host species had notably longer branches than the others, two of which were closely related to reference strain Res 4.1, an isolate from the light organ of *S. tubifer* collected in Okinawa, Japan in 2014. Corresponding with longer branch lengths, these four symbionts had more than 3 times as many SNPs than any other sample, ranging between 66,583 and 72,219 SNPs (Supplementary Table 1). Interestingly, these four specimens were collected from two locations, Sydney, Australia and Kochi, Japan, which had the lowest minimum annual temperatures of all collection sites in this study (Table 1). Furthermore, there were 20,082 SNPs in common among these samples that were not present in the core set of SNPs identified across all samples.

### Analysis of Co-divergence

**FIGURE 5 |:**
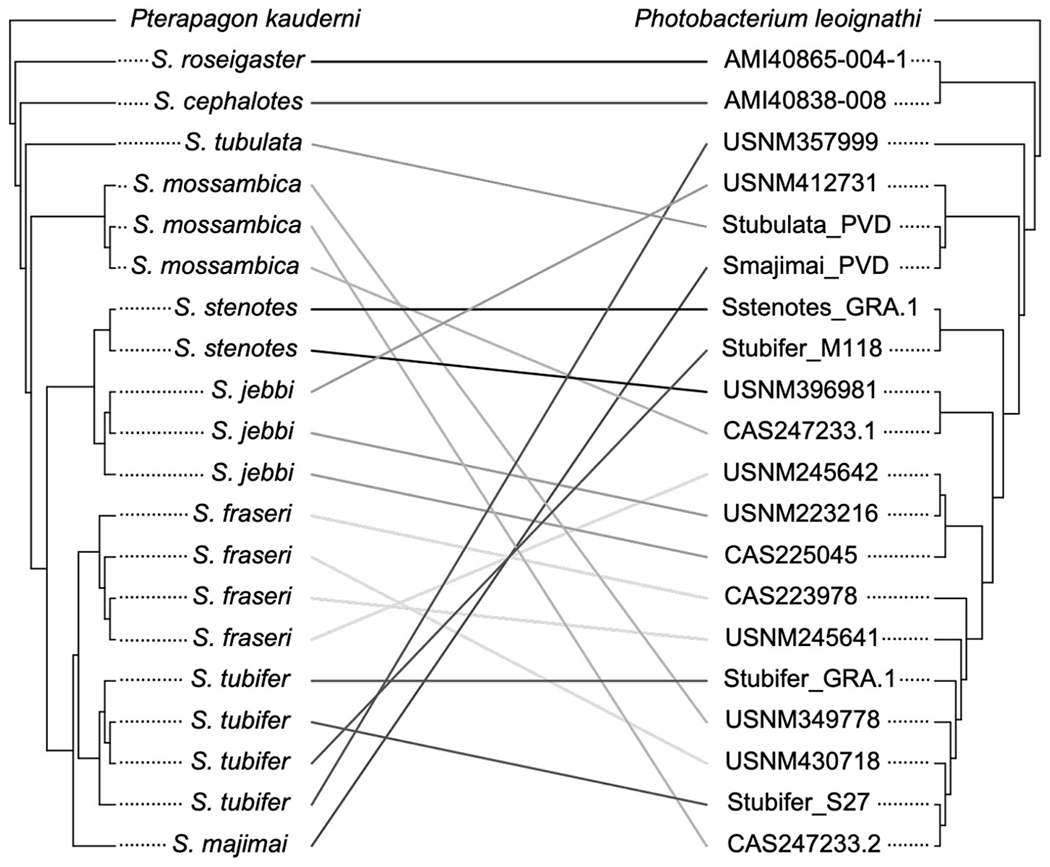
Analysis of the phylogenetic relationships of *Siphamia* hosts (left) and their light organ symbionts (right) revealed no evidence of co-divergence. The host cladogram is based on *COI* gene sequences and the symbiont cladogram is based on a core set of 1,471 single nucleotide polymorphisms. Linkages between individual hosts and their symbionts are shown and colored according to host species.

Twenty specimens had informative sequence information for both the host and symbiont, and thus, we were able to carry out an analysis of co-divergence based on the host *COI* phylogeny and corresponding symbiont phylogeny for these individuals. This analysis revealed no evidence of co-divergence of *Siphamia* hosts and their light organ symbionts (*P* = 0.13) as seen in Figure 5. However, *S. roseigaster* and *S. cephalotes* fall out as sister lineages relative to the rest of *Siphamia*, and their symbionts follow a similar pattern, forming a sister clade to the rest of *P. mandapamensis* Clade II.

## DISCUSSION

Our results indicate that the symbiosis between cardinalfishes in the genus *Siphamia* and the luminous bacterium *Photobacterium mandapamensis* is highly conserved across host species, over geographic space, and over decades. All of the symbionts for which we recovered enough sequence data to analyze were identified as members of Clade II of *P. mandapamensis* (Kaeding et al., 2007). The subspecies level of symbiont specificity observed between *Siphamia* species and *P. mandapamensis* Clade II over the broad geographic and temporal ranges examined is surprising, especially given that the bacteria is a facultative symbiont. Such a high degree of specificity is expected for vertically transmitted symbioses in which a host transfers its symbiotic bacteria directly to its offspring (Moran, 2006), but is unexpected for environmentally transmitted symbioses where symbionts are acquired from a genetically diverse, free-living population of bacteria by each new host generation. This degree of specificity across the *Siphamia* genus is higher than has been documented for other symbiotically luminous fishes; other genera typically associate with a few different species of luminous bacteria, and two symbiont species have even been described cohabiting within the same light organ (Kaeding et al., 2007). Similarly, within genera of sepiolid and loliginid squids, including *Euprymna*, hosts associate with several different species of bioluminescent bacteria (Guerrero-Ferreira and Nishiguchi, 2007), further highlighting the markedly higher subspecies-level of symbiont specificity observed throughout the *Siphamia* genus. This highly conserved relationship between *Siphamia* and *P. mandapamensis* Clade II suggests that there are attributes of this clade of bacteria that are essential for the symbiosis as well as selection mechanisms that help each new host generation maintain specificity.

*Siphamia tubifer* larvae only take up symbionts in their pelagic phase, at least 1 week after hatching, when their light organ becomes receptive to colonization (Dunlap et al., 2012). Yet, *Photobacterium* spp. normally occurs in relatively low concentrations in the pelagic environment (Troussellier et al., 2017), and even more so at the sub-species level. For a larval host to rely on this improbable encounter in the open water would be considered a very risky strategy. However, it has been shown that *S. tubifer* hosts regularly excrete their luminous symbiont with fecal waste (Dunlap and Nakamura, 2011), thereby enriching its population in the immediate environment. Indeed, a previous study of *S. tubifer* symbiont genomics revealed fine-scale population structure of *P. mandapamensis* Clade II among geographic locations, indicating that symbiont populations are heavily influenced by their local hosts (Gould and Dunlap, 2019), a mechanism that has also been documented for the bioluminescent squid *Euprymna scolopes* and its luminous symbiont, *Aliivibrio fischeri* (Lee and Ruby, 1994; Wollenberg and Ruby, 2009), although squid hosts can acquire a luminous symbiont immediately upon hatching (Nyholm and McFall-Ngai, 2004). The specific timing of symbiont acquisition for *S. tubifer* larvae in the wild has yet to be defined, but this local enrichment may be a key factor in mitigating the risk of relying on environmental transmission of a narrow range of luminous bacteria, and for *Siphamia* hosts, ensures that *P. mandapamensis* Clade II will be readily available to new recruits anywhere that populations of *Siphamia* already occur.

The apparent preference to associate with *P. mandapamensis* Clade II over strains in Clade I also suggests that there are critical strain-level differences between members of these clades that may be of consequence to the host. However, most studies of microbial symbioses overlook subspecies-level symbiont variation, even though this variation can have important impacts on a host. For example, patterns of strain variation in *A. fischeri* have been observed within and between populations of their host squid, *E. scolopes* (Jones et al., 2006; Wollenberg and Ruby, 2009), and different strains have different colonization efficiencies (Lee and Ruby, 1994; Bongrand et al., 2016), mechanisms of biofilm formation during host colonization (Rotman et al., 2019), and could have variable fitness consequences to their host (Koch et al., 2014). In this study we characterized strain variation in *P. mandapamensis* associated with various *Siphamia* hosts but saw no distinct correlation between symbiont strain and host species with respect to time or geography. However, we did observe some strain divergence associated with colder temperatures. Four of the *Siphamia* specimens examined had more than three times as many symbiont SNPs as the others. These four individuals were all collected from more temperate regions in Japan and Australia, which had the lowest minimum annual temperatures of all locations surveyed (Table 1). Temperature is a driving factor of the distribution of bacteria in the marine environment (Sul et al., 2013), and has been shown to influence the symbiotic associations of other marine taxa, such as sepiolid squids (Nishiguchi, 2000; Zamborsky and Nishiguchi, 2011) and cnidarians (Herrera et al., 2020). Thus, the symbionts associated with these four specimens could have genetic adaptations to slightly cooler temperatures. Future studies investigating the influence of temperature on strain diversity and host colonization efficiency would help to elucidate the role that temperature might play in the *Siphamia-Photobacterium mandapamensis* symbiosis.

Our primary objective of this study was to sequence the symbionts found in the light organs of various *Siphamia* species, but we were able to recover enough host sequence data to also construct a reasonably well-supported host phylogeny. This allowed us to examine codivergence of hosts and their microbial symbionts. Although we found no evidence of codivergence, the high degree of specificity maintained for this symbiosis across host species over a broad geographic range suggests that this association is genetically constrained by the host. This host-mediated selection poses the question of whether strains in Clade II of *P. mandapamensis* provide a fitness advantage to the host compared to other bacteria moving through the gut of *Siphamia*, including *P. mandapamensis* Clade I and other luminous bacteria. Understanding the genetic architecture of this symbiont selection mechanism may be key to deciphering the highly specific nature of the association. It should be noted that a lack of co-diversification does not preclude a history of coevolution of host and symbiont in the system (Moran, 2006), and members of Clade II of *P. mandapamensis* are likely to have specific adaptations that provide them with a fitness advantage inside the light organ environment of *Siphamia* hosts.

*Siphamia*, the only symbiotically luminous genus of cardinalfish, is monophyletic and divergent from the rest of the Apogonidae (Thacker and Roje, 2009). The absence of this symbiosis in all other cardinalfish genera, including the other bioluminescent genera, brings up intriguing questions regarding the role of the symbiosis in the evolution of the *Siphamia* genus, specifically whether the ancestral *Siphamia* evolved via speciation by symbiosis (Wallin, 1927), and whether this association has since acted as a key innovation for the genus. Key innovations help clades persist and diversify by shifting them into a new adaptive zone, increasing fitness, or increasing the propensity for specialization (Heard and Hauser, 1995). In *Siphamia*, the association with luminous bacteria is thought to provide a fitness benefit either by protecting against predation via counter-illumination or by attracting prey while foraging at night. This fitness advantage could have helped *Siphamia* persist over evolutionary time, allowing it to diversify. Symbioses have been credited for catalyzing or enhancing adaptive radiations in several systems, such as symbioses with photosynthetic protists in foraminifera (Norris, 1996) and heterobranchia (Wägele, 2004), and symbioses between gall-forming insects and fungi (Joy, 2013).

This study also highlights the potential for formalin-fixed, fluid-preserved museum specimens to be used to investigate microbial symbioses. Adapting recently-developed molecular techniques to extract and prepare DNA from these specimens for sequencing, including the use of single-stranded DNA as templates to construct sequence libraries, we recovered informative sequence data for both the host and its bacterial symbiont. This method allowed us to identify and compare strain-level differences between the bacterial symbionts of many host species collected across dates spanning nearly a century and dispersed widely throughout the Indo-Pacific. However, a limitation of working with formalin-fixed museum specimens is the fragmented nature of the resulting sequence data, which required us to use consensus sequences to characterize the light organ symbionts. Thus, we recognize that our methods were not designed to detect rare symbiont strains. Additional strains, including other *Photobacterium* species, may have also been present at low abundance but could not be characterized with this approach. However, we are confident that *P. mandapamensis* Clade II is the most abundant symbiont in all of our *Siphamia* specimens, and if any alternative symbionts had a comparable abundance, they would have been detected by our SNP analysis and fallen outside of *P. mandapamensis* Clade II in the phylogenetic tree. Additional investigations using fresh specimens and culture-based methods would be helpful to corroborate our results. Notably, we also saw no clear correlation between sequence quality or yield and variables such as specimen age, size, or DNA input. Initial specimen preservation methods and long-term storage conditions likely contributed more to the variability in DNA quality (Hykin et al., 2015), and this specimen metadata is often lacking. Nevertheless, we were successful at retrieving genetic information from decades old specimens initially fixed in formalin that enabled us to characterize the specificity of the bioluminescent symbiosis across the *Siphamia* genus throughout the Indo-Pacific. With the continued advancement of genomic techniques and sequencing technologies, our ability to retrieve genetic information for both hosts and bacterial symbionts from museum specimens will further our understanding of these critical associations.

## Supplementary Material

GouldEtal2021_SuppMat

## Figures and Tables

**TABLE 1 |:** Information for the *Siphamia* specimens sampled in this study.

Specimen ID	Species	Location	Latitude	Longitude	Year	Min Temp	Max Temp
AMI18353-041	*jebbi*	Fiji	−18.1	178.4	1974	31.06	25.57
AMI18740-066	*jebbi*	Australia	−14.6	145.6	1975	29.48	24.69
AMI19450-018	*tubifer*	Australia	−14.7	145.5	1975	29.94	24.09
AMI20353-001	*majimai*	Australia	−20.5	116.8	1972	31.76	22.61
AMI20753-031	*tubulata*	Australia	−14.6	145.2	1979	30.04	23.54
AMI33715-016	*jebbi*	Australia	−10.4	144.4	1993	29.70	25.08
AMI37933-007	*tubifer*	Vanuatu	−13.8	167.4	1997	30.18	27.30
AMI40838-008	*cephalotes*	Australia	−33.8	151.2	2001	23.03	15.49
AMI40865-004	*roseigaster*	Australia	−33.8	151.2	2001	24.16	18.64
AMIB4208	*majimai*	Australia	−23.4	151.9	1958	28.11	21.64
AMIB4247	*tubifer*	Vanuatu	−16.5	167.4	1959	29.64	26.23
CAS247233	*mossambica*	Zanzibar	−6.2	39.2	2018	31.06	25.92
CAS222309	*jebbi*	Fiji	−16.8	178.3	2002	30.28	26.16
CAS223855	*jebbi*	Fiji	−18.2	178.4	2002	29.88	25.89
CAS223939	*jebbi*	Fiji	−18.1	178.4	2002	29.88	25.89
CAS223978	*unknown*	Fiji	−18.1	178.4	2002	29.88	25.89
CAS223979	*fraseri*	Fiji	−18.1	178.4	2002	29.88	25.89
CAS225045	*jebbi*	Fiji	−18.1	178.4	1999	29.88	25.89
CAS27441	*tubifer*	Philippines	10.2	123.8	1931	30.77	27.89
CAS28515	*tubulata*	Australia	−14.2	144.3	1973	30.31	23.60
CAS84356	*tubifer*	Palau	7.4	134.4	2012	30.33	28.57
Stubifer_M118	*tubifer*	Ryukyu Islands	26.7	127.9	2013	29.60	20.83
Smajimai_PVD	*majimai*	Japan	32.8	133.5	2007	28.75	18.68
Stubulata_PVD	*tubulata*	Japan	32.7	132.6	2007	28.28	18.02
Stubifer_S27	*tubifer*	Ryukyu Islands	26.7	127.9	2013	29.60	20.83
Sstenotes_GRA	*stenotes*	Indonesia	−3.9	134	2006	30.80	26.42
Stubifer_GRA	*tubifer*	Indonesia	−3.7	133.7	2006	30.88	26.60
USNM112099	*elongata*	Philippines	16.9	120.2	1909	30.74	27.58
USNM142281	*fuscolineata*	Marshall Islands	11.6	165.4	1946	29.70	27.05
USNM203781	*corallicola*	Borneo	6	116.1	1965	31.33	28.34
USNM223216	*jebbi*	Micronesia	6.9	158.1	1980	30.73	28.31
USNM245638	*jebbi*	Fiji	−19.2	179.8	1982	29.10	25.04
USNM245641	*fraseri*	Fiji	−20.6	−178.7	1982	28.60	24.12
USNM245642	*fraseri*	Fiji	−21.7	−178.6	1982	28.08	23.32
USNM298542	*brevilux*	Papua New Guinea	−5.2	145.8	1988	30.83	28.73
USNM341594	*jebbi*	Tonga	−18.7	−174.1	1993	29.02	25.28
USNM341595	*tubifer*	Tonga	−18.7	−174	1993	29.59	25.63
USNM349778	*mossambica*	Mauritius	−20.2	57.4	1995	28.76	23.68
USNM357884	*tubifer*	Philippines	16.4	119.9	1980	30.60	27.53
USNM357889	*spinicola*	Papua New Guinea	−10.6	151	1975	29.83	25.76
USNM357892	*tubifer*	Red Sea	29.5	34.9	1969	28.10	21.47
USNM357897	*tubifer*	Andaman Islands	11.8	92.9	1963	31.15	27.89
USNM357999	*tubifer*	Sri Lanka	8.6	81.2	1970	30.58	27.33
USNM358001	*majimai*	Philippines	9.4	123.3	1978	30.06	27.19
USNM374480	*majimai*	Australia	−23.9	152.4	1966	27.85	21.93
USNM374837	*tubifer*	Wallis and Futuna	−13.3	−176.1	2000	30.27	28.26
USNM396981	*stenotes*	Indonesia	−4	134.4	2006	31.57	27.68
USNM412731	*jebbi*	Philippines	12.7	120.5	2003	30.79	28.54
USNM430718	*fraseri*	French Polynesia	−22.6	−152.8	2013	27.80	23.49

Listed are each specimen lot’s catalog number or unique identifier, species identification, sampling information including location, latitude, longitude, year, the minimum and maximum temperatures at that location, and the standard length of the individual sampled. Sea surface temperatures from the topmost meter of water at the geographical point of specimen collection were calculated as the temporal minimum and maximum from monthly climatologies (2002–2009) extracted from the Aqua-MODIS database available on Bio-ORACLE (Tyberghein et al., 2012).

**TABLE 2 |:** Average nucleotide identities (% ANI) of the consensus sequences of the light organ symbionts from the *Siphamia* specimens sampled in this study relative to several symbiotic *Photobacterium* species: *P. kishitanii* (pjapo1.1), *P. leiognathi* (lrivu4.1), *P. mandapamensis* (*P. mand*.), Clade I (ajapo4.1), and Clade II (gjord1.1, svers1.1).

Specimen ID	*P. kishitanii* (pjapo1.1)		*P. leiognathi* (lrivu4.1)		*P. mand*. Clade I (ajapo4.1)		*P. mand*. Clade II (gjord1.1)		*P. mand*. Clade II (*svers*1.1)		%10x coverage

	% ANI	% ortholog	% ANI	% ortholog	% ANI	% ortholog	% ANI	% ortholog	% ANI	% ortholog	
AMI18353-041	79.4	35.2	91.1	84.8	**94.8**	88.0	**95.5**	94.6	**95.5**	97.8	2.6
AMI18740-066	80.4	10.8	83.3	30.3	86.8	41.7	84.8	59.2	85.4	62.3	2.1
AMI19450-018.1	78.1	36.4	89.4	82.4	93.1	84.3	93.6	91.7	94	95.5	2.6
AMI19450-018.2	79.4	35.8	91.1	85.4	**94.7**	88.6	**95.4**	94.7	**95.4**	98.3	45.3
AMI20353-001	77.5	38.3	87.8	79.2	91.8	81.1	92.1	89.5	92.6	92.1	2.3
AMI20753-031	79.6	32.8	90.7	85.1	94.3	88.1	**94.9**	94.5	**95.1**	97.9	14.9
AMI33715-016	79.7	33.6	91.2	84.9	**94.9**	88.6	**95.5**	94.2	**95.6**	98.0	13.7
AMI37933-007	79.5	30.7	90.2	83.7	94	87.3	**94.6**	93.1	**95**	97.4	1.9
AMI40838-008	80.2	39.2	92.8	85.8	**96.5**	89.4	**97.2**	94.8	**97.1**	98.8	92.3
AMI40865-004.1	80.1	39.5	92.9	85.5	**96.5**	89.5	**97.1**	95.0	**97.2**	98.4	93.4
AMI40865-004.2	79.7	37.9	92	84.0	**95.7**	87.2	**96**	92.5	**96.2**	95.9	5.9
AMIB4208	80.7	17.7	90.3	84.3	94	87.9	**94.6**	93.9	90	72.1	6.7
AMIB4247	79.7	35.0	91.2	84.9	**94.8**	88.5	**95.5**	94.6	**95.7**	97.9	9.3
CAS222309	79.9	34.7	91.3	84.9	**95**	89.1	**95.7**	94.3	**95.8**	98.0	95.2
CAS223855	79.4	33.9	91	84.8	**94.7**	87.6	**95.3**	94.4	**95.5**	97.6	6.8
CAS223939	79.8	35.3	91.3	85.1	**95**	88.4	93.2	90.2	**95.8**	97.6	12
CAS223978	79.9	33.9	91	85.3	**94.6**	88.5	88.6	42.1	**95.4**	98.0	95.4
CAS223979	78.9	30.3	89.5	84.1	93.3	87.1	93.9	93.1	94.2	97.2	17.7
CAS225045	80.1	33.4	91	84.4	**94.7**	88.2	**95.3**	94.5	**95.3**	98.0	96.1
CAS247233.1	80	37.5	92	86.1	**95.8**	89.6	**96.6**	95.1	**97**	97.6	76.9
CAS247233.2	79.9	34.7	91.1	85.1	**94.7**	88.2	**95.4**	94.6	**95.5**	98.5	96.4
CAS27441	80	36.4	91.8	86.0	**95.5**	89.5	**96.2**	95.7	**96.6**	98.2	95.3
CAS28515	79.6	33.9	91.1	84.7	**94.9**	88.7	**95.5**	94.3	**95.6**	98.1	5.6
CAS84356	80	37.1	92.1	85.2	**96**	89.1	**96.6**	95.1	**97.1**	97.4	64.8
Smajimai_PVD	80.1	39.6	92.8	85.2	**96.7**	89.4	**97.4**	94.5	**97.3**	98.4	94.8
Sstenotes_GRA.1	80.1	35.5	91.8	85.1	**95.4**	88.6	**96.1**	94.7	**96.2**	98.0	95.8
Sstenotes_GRA.2	79.6	38.1	91.6	84.8	**95.2**	87.8	**95.9**	93.9	**96.5**	95.8	0.5
Stubifer_GRA.1	79.9	34.6	91.1	84.6	**94.7**	88.4	**95.4**	94.4	**95.5**	97.9	95.8
Stubifer_GRA.2	79.8	39.0	92.2	86.2	**96**	90.0	**96.8**	95.7	**97.2**	97.9	52
Stubifer_M118	80	33.2	91	84.7	**94.6**	88.1	**95.3**	94.6	**95.3**	98.2	96.2
Stubifer_S27	80	37.0	92.1	86.2	**95.7**	89.3	**96.5**	95.7	**96.9**	97.6	88.9
Stubulata_PVD	80.1	39.8	92.9	85.1	**96.7**	89.3	**97.4**	95.0	**97.4**	98.2	95.1
USNM112099	78	38.1	89.3	81.9	93.3	85.0	93.8	91.3	94.1	95.5	3.7
USNM142281	79.7	31.1	91.8	84.5	**95.7**	87.6	**96.3**	93.6	**94.6**	96.9	96.1
USNM203781	79.6	34.9	91.2	84.5	**95**	88.4	**95.6**	94.7	**95.6**	98.2	2.5
USNM223216	80	33.8	91.3	85.3	**94.8**	88.4	**95.5**	94.4	**95.6**	98.2	96.5
USNM245638	79.7	35.4	91.4	84.9	**95.3**	89.6	**96**	94.6	**95.9**	98.0	35.6
USNM245641	79.9	35.1	91.4	85.0	**95.1**	88.9	**95.7**	94.5	**95.9**	98.6	97.5
USNM245642	79.9	35.6	91.4	85.3	**95.1**	89.0	**95.7**	94.6	**95.8**	98.1	96.2
USNM298542	78.6	33.8	89.4	84.0	93.3	86.1	93.7	93.1	94	95.7	0.1
USNM341594	79.8	33.6	90.9	84.7	**94.6**	88.6	**95.2**	94.6	**95.3**	98.1	61.4
USNM341595	79.8	32.4	90.5	84.7	94	87.8	**94.6**	93.9	**94.7**	97.8	95.5
USNM349778	79.9	33.7	91	85.0	**94.6**	88.5	**95.2**	94.4	**95.3**	98.3	95.8
USNM357884	79.6	33.3	90.8	84.9	**94.5**	88.4	**95.2**	94.6	**95.5**	97.6	33.3
USNM357889	79.5	34.1	90.8	85.1	**94.5**	88.3	**95.1**	94.6	**95.3**	98.2	42.7
USNM357892	79.6	33.9	91	85.3	**94.7**	88.9	**95.4**	94.4	**95.6**	98.3	3.9
USNM357897	79.4	29.8	89.4	84.3	93	87.8	93.5	94.2	93.7	97.6	95
USNM357999	79.9	34.7	91.2	85.1	**94.9**	88.7	**95.5**	94.6	**95.6**	98.4	95.6
USNM358001	79.3	33.6	90.6	84.6	**94.5**	88.2	**95.1**	94.1	**95.3**	98.0	3.2
USNM374480	79.6	32.1	90.5	84.7	94	88.0	**94.6**	94.4	**94.8**	98.0	40.5
USNM374837	79.7	34.9	91.2	84.9	**94.9**	88.3	**95.6**	94.5	**95.7**	97.8	22.8
USNM396981	79.9	34.3	90.9	85.4	**94.6**	88.3	**95.2**	94.3	**95.3**	98.1	96.1
USNM412731	80.2	35.8	91.9	85.5	**95.6**	89.3	**96.3**	94.9	**96.4**	98.3	95.9
USNM430718	79.8	34.3	91	85.0	**94.5**	88.3	**95.2**	94.4	**95.3**	98.1	95.3

Values in bold are 94.5% or greater. Also listed is each specimen’s catalog number or unique identifier, the percent of sequence fragments that were identified as orthologous matches (% ortholog) by fastANI (Jain et al., 2018), and each symbiont’s percent genome coverage at 10x sequencing depth.

## Data Availability

The datasets presented in this study can be found in online repositories. The names of the repository/repositories and accession number(s) can be found below: https://www.ncbi.nlm.nih.gov/, SAMN17043373-SAMN17043448.

## References

[R1] Allio R, Schomaker-Bastos A, Romiguier J, Prosdocimi F, Nabholz B, and Delsuc F (2020). MitoFinder: efficient automated large-scale extraction of mitogenomic data in target enrichment phylogenomics. Mol. Ecol. Resour 20, 892–905. doi: 10.1111/1755-0998.13160 32243090 PMC7497042

[R2] Altschul SF, Gish W, Miller W, Myers EW, and Lipman DJ (1990). Basic local alignment search tool. J. Mol. Biol 215, 403–410. doi: 10.1016/S0022-2836(05)80360-2 2231712

[R3] Ast JC, and Dunlap PV (2004). Phylogenetic analysis of the lux operon distinguishes two evolutionarily distinct clades of *Photobacterium leiognathi*. Arch. Microbiol 181, 352–361. doi: 10.1007/s00203-004-0663-7 15034641

[R4] Baker LJ, Freed LL, Easson CG, Lopez JV, Fenolio D, Sutton TT, (2019). Diverse deep-sea anglerfishes share a genetically reduced luminous symbiont that is acquired from the environment. eLife. 8:e47606. doi: 10.7554/eLife.47606 31571583 PMC6773444

[R5] Bolger AM, Lohse M, and Usadel B (2014). Trimmomatic: a flexible trimmer for Illumina sequence data. Bioinformatics 30, 2114–2120. doi: 10.1093/bioinformatics/btu170 24695404 PMC4103590

[R6] Bongrand C, Koch EJ, Moriano-Gutierrez S, Cordero OX, McFall-Ngai M, Polz MF, (2016). A genomic comparison of 13 symbiotic Vibrio fischeri isolates from the perspective of their host source and colonization behavior. ISME J. 10, 2907–2917. doi: 10.1038/ismej.2016.69 27128997 PMC5148191

[R7] Bright M, and Bulgheresi S (2010). A complex journey: transmission of microbial symbionts. Nat. Rev. Microbiol 8, 218–230. doi: 10.1038/nrmicro2262 20157340 PMC2967712

[R8] Brown MV, Lauro FM, DeMaere MZ, Muir L, Wilkins D, Thomas T, (2012). Global biogeography of SAR11 marine bacteria. Mol. Syst. Biol 8:595. doi: 10.1038/msb.2012.28 22806143 PMC3421443

[R9] Cingolani P, Platts A, Wang LL, Coon M, Nguyen T, Wang L, (2012). A program for annotating and predicting the effects of single nucleotide polymorphisms, SnpEff: SNPs in the genome of Drosophila melanogaster strain w1118; iso-2; iso-3. Fly. 6, 80–92. doi: 10.4161/fly.19695 22728672 PMC3679285

[R10] Darling AE, Jospin G, Lowe E, Matsen FA, Bik HM, and Eisen JA (2014). PhyloSift: phylogenetic analysis of genomes and metagenomes. PeerJ. 2:e243. doi: 10.7717/peerj.243 24482762 PMC3897386

[R11] Davis MP, Sparks JS, and Smith WL (2016). Repeated and widespread evolution of bioluminescence in marine fishes. PLoS ONE 11:0155154. doi: 10.1371/journal.pone.0155154 PMC4898709 27276229

[R12] Dunlap PV, Ast JC, Kimura S, Fukui A, Yoshino T, and Endo H (2007). Phylogenetic analysis of host-symbiont specificity and codivergence in bioluminescent symbioses. Cladistics 23, 507–532. doi: 10.1111/j.1096-0031.2007.00157.x

[R13] Dunlap PV, Gould AL, Wittenrich ML, and Nakamura M (2012). Symbiosis initiation in the bacterially luminous sea urchin cardinalfish *Siphamia versicolor*. J. Fish Biol 81, 1340–1356. doi: 10.1111/j.1095-8649.2012.03415.x 22957874

[R14] Dunlap PV, and Nakamura M (2011). Functional morphology of the luminescence system of *Siphamia versicolor* (Perciformes: Apogonidae), a bacterially luminous coral reef fish. J. Morphol 272, 897–909. doi: 10.1002/jmor.10956 21541984

[R15] Dunlap PV, and Urbanczyk H (2013). “Luminous bacteria BT - the prokaryotes: prokaryotic physiology and biochemistry,” in The Prokaryotes: Prokaryotic Physiology and Biochemistry, eds Rosenberg E, DeLong EF, Lory S, Stackebrandt E, and Thompson F (Berlin: Springer Berlin Heidelberg).

[R16] Edgar RC (2004). MUSCLE: Multiple sequence alignment with high accuracy and high throughput. Nucleic Acids Res. 32, 1792–1797. doi: 10.1093/nar/gkh340 15034147 PMC390337

[R17] Gon O, and Allen GR (2012). Revision of the Indo-Pacific cardinalfish genus Siphamia (Perciformes: Apogonidae). Zootaxa 3294, 1–84. doi: 10.11646/zootaxa.3294.1.1 25543639

[R18] Goris J, Konstantinidis KT, Klappenbach JA, Coenye T, Vandamme P, and Tiedje JM (2007). DNA–DNA hybridization values and their relationship to whole-genome sequence similarities. Int. J. Syst. Evol. Microbiol 57, 81–91. doi: 10.1099/ijs.0.64483-0 17220447

[R19] Gould AL, Dougan KE, Koenigbauer ST, and Dunlap PV (2016). Life history of the symbiotically luminous cardinalfish Siphamia tubifer (Perciformes: Apogonidae). J. Fish Biol 89, 1359–1377. 27329350 10.1111/jfb.13063

[R20] Gould AL, and Dunlap PV (2019). Shedding light on specificity: population genomic structure of a symbiosis between a coral reef fish and luminous bacterium. Front. Microbiol 10:2670. doi: 10.3389/fmicb.2019.02670 31824455 PMC6879551

[R21] Guerrero-Ferreira RC, and Nishiguchi MK (2007). Biodiversity among luminescent symbionts from squid of the genera *Uroteuthis*, *Loliolus* and *Euprymna* (Mollusca: Cephalopoda). Cladistics 23, 497–506. doi: 10.1111/j.1096-0031.2007.00155.x 22707847 PMC3374722

[R22] Heard SB, and Hauser DL (1995). Key evolutionary innovations and their ecological mechanisms. Hist. Biol 10, 151–173. doi: 10.1080/10292389509380518

[R23] Hendry TA, de Wet JR, and Dunlap PV (2014). Genomic signatures of obligate host dependence in the luminous bacterial symbiont of a vertebrate. Environ. Microbiol 16, 2611–2622. doi: 10.1111/1462-2920.12302 24118864

[R24] Hendry TA, Freed LL, Fader D, Fenolio D, Sutton TT, and Lopez JV (2018). Ongoing transposon-mediated genome reduction in the luminous bacterial symbionts of deep-sea ceratioid anglerfishes. MBio 9:e01017–17. doi: 10.1128/mBio.01033-18 29946051 PMC6020299

[R25] Herrera M, Klein SG, Campana S, Chen JE, Prasanna A, Duarte CM, (2020). Temperature transcends partner specificity in the symbiosis establishment of a cnidarian. ISME J. 1013:1–13. doi: 10.1038/s41396-020-00768-y PMC7852570 32934356

[R26] Hykin SM, Bi K, and McGuire JA (2015). Fixing formalin: a method to recover genomic-scale DNA sequence data from formalin-fixed museum specimens using high-throughput sequencing. PLoS ONE 10:e0141579. doi: 10.1371/journal.pone.0141579 26505622 PMC4623518

[R27] Jain C, Rodriguez-R,LM, Phillippy AM, Konstantinidis KT, and Aluru S (2018). High throughput ANI analysis of 90K prokaryotic genomes reveals clear species boundaries. Nat. Commun 9:5114. doi: 10.1038/s41467-018-07641-9 30504855 PMC6269478

[R28] Jombart T, Balloux F, and Dray S (2010). adephylo: exploratory analyses for the phylogenetic comparative method. Bioinformatics 26, 1–21. doi: 10.1093/bioinformatics/btq292 20525823

[R29] Jones BW, Lopez JE, Huttenburg J, and Nishiguchi MK (2006). Population structure between environmentally transmitted vibrios and bobtail squids using nested clade analysis. Mol. Ecol 15, 4317–4329. doi: 10.1111/j.1365-294X.2006.03073.x 17107468

[R30] Joy JB (2013). Symbiosis catalyses niche expansion and diversification. Proc. R. Soc. B Biol. Sci 280:20122820. doi: 10.1098/rspb.2012.2820 PMC3574373 23390106

[R31] Kaeding AJ, Ast JC, Pearce MM, Urbanczyk H, Kimura S, Endo H, (2007). Phylogenetic diversity and cosymbiosis in the bioluminescent symbioses of “Photobacterium mandapamensis”. Appl. Environ. Microbiol 73, 3173–3182. doi: 10.1128/AEM.02212-06 17369329 PMC1907103

[R32] Koch EJ, Miyashiro T, McFall-Ngai MJ, and Ruby EG (2014). Features governing symbiont persistence in the squid–vibrio association. Mol. Ecol 23, 1624–1634. doi: 10.1111/mec.12474 24118200 PMC3907463

[R33] Kozlov AM, Darriba D, Flouri T, Morel B, and Stamatakis A (2019). RAxML-NG: a fast, scalable and user-friendly tool for maximum likelihood phylogenetic inference. Bioinformatics 35, 4453–4455. doi: 10.1093/bioinformatics/btz305 31070718 PMC6821337

[R34] Lee KH, and Ruby EG (1994). Effect of the squid host on the abundance and distribution of symbiotic *Vibrio fischeri* in nature. Appl. Environ. Microbiol 60, 1565–1571. doi: 10.1128/AEM.60.5.1565-1571.1994 16349257 PMC201518

[R35] Li H (2013). Aligning Sequence Reads, Clone Sequences and Assembly Contigs With BWA-MEM. arXiv preprint arXiv:1303.3997 http://arxiv.org/abs/1303.3997

[R36] Li H, Handsaker B, Wysoker A, Fennell T, Ruan J, Homer N, (2009). 1000 genome project data., Abecasis G, Durbin R; 1000 Genome Project Data 000 Genome Project Data 000 Genome Project Data Processing Subgroup. The Sequence alignment/map (SAM) format and SAMtools. Bioinformatics 25, 2078–2079. doi: 10.1093/bioinformatics/btp352 19505943 PMC2723002

[R37] Matias AM, and Hereward J (2018). The complete mitochondrial genome of the five-lined cardinalfish *Cheilodipterus quinquelineatus* (Apogonidae). Mitochondrial DNA Part B, 3, 521–522. doi: 10.1080/23802359.2018.1467221 33474225 PMC7800949

[R38] Moran NA (2006). Symbiosis. Curr. Biol 16, 866–871. doi: 10.1016/j.cub.2006.09.019 17055966

[R39] Nguyen LT, Schmidt HA, Von Haeseler A, and Minh BQ (2015). IQ-TREE: a fast and effective stochastic algorithm for estimating maximum-likelihood phylogenies. Mol. Biol. Evol, 32, 268–274. doi: 10.1093/molbev/msu300 25371430 PMC4271533

[R40] Nishiguchi MK (2000). Temperature affects species distribution in symbiotic populations of Vibrio spp. Appl. Environ. Microbiol 66, 3550–3555. doi: 10.1128/AEM.66.8.3550-3555.2000 10919820 PMC92184

[R41] Nishiguchi MK, and Nair VS (2003). Evolution of symbiosis in the Vibrionaceae: a combined approach using molecules and physiology. Int. J. Syst. Evol. Microbiol 53, 2019–2026. doi: 10.1099/ijs.0.02792-0 14657139

[R42] Norris RD (1996). Symbiosis as an evolutionary innovation in the radiation of Paleocene planktic foraminifera. Paleobiology 22, 461–480. doi: 10.1017/S0094837300016468

[R43] Norsworthy AN, and Visick KL (2013). Gimme shelter: how *Vibrio fischeri* successfully navigates an animal’s multiple environments. Front. Microbiol 4, 356. doi: 10.3389/fmicb.2013.00356 24348467 PMC3843225

[R44] Nyholm SV, and McFall-Ngai M (2004). The winnowing: establishing the squid–Vibrio symbiosis. Nat. Rev. Microbiol 2, 632–642. doi: 10.1038/nrmicro957 15263898

[R45] Padgham M, and Sumner MD (2020). *geodist: Fast, Dependency-Free Geodesic Distance Calculations*. R package version 0.0.4. Available online at: https://CRAN.R-project.org/package=geodist

[R46] Portik DM and Wiens JJ (2020). SuperCRUNCH: A bioinformatics toolkit for creating and manipulating supermatrices and other large phylogenetic datasets. Methods Ecol. Evol 11, 763–772. doi: 10.1111/2041-210X.13392

[R47] Reichelt JL, Nealson K, and Hastings JW (1977). The specificity of symbiosis: pony fish and luminescent bacteria. Arch. Microbiol 112, 157–161. doi: 10.1007/BF00429329

[R48] Revell L (2011). Phytools: An R package for phylogenetic comparative biology (and other things). Methods Ecol. Evol 3, 217–223. doi: 10.1111/j.2041-210X.2011.00169.x

[R49] Ronquist F (1998). Phylogenetic approaches in coevolution and biogeography. Zool. Scr 26, 313–322. doi: 10.1111/j.1463-6409.1997.tb00421.x

[R50] Rotman ER, Bultman KM, Brooks JF, Gyllborg MC, Burgos HL, Wollenberg MS, (2019). Natural strain variation reveals diverse biofilm regulation in squid-colonizing Vibrio fischeri. J. Bacteriol 201:e00033–19. doi: 10.1128/JB.00033-19 30782630 PMC6456852

[R51] Ruane S, and Austin CC (2017). Phylogenomics using formalin-fixed and 100+ year-old intractable natural history specimens. Mol. Ecol. Resources, 17, 1003–1008. doi: 10.1111/1755-0998.12655 28118515

[R52] Seemann T (2015). Snippy: Fast Bacterial Variant Calling From NGS Reads. Snippy: fast bacterial variant calling from NGS reads.

[R53] Sul WJ, Oliver TA, Ducklow HW, Amaral-Zettler LA, and Sogin ML (2013). Marine bacteria exhibit a bipolar distribution. Proc. Natl. Acad. Sci. U.S.A 110, 2342–2347. doi: 10.1073/pnas.1212424110 23324742 PMC3568360

[R54] Thacker CE, and Roje DM (2009). Phylogeny of cardinalfishes (Teleostei: Gobiiformes: Apogonidae) and the evolution of visceral bioluminescence. Mol. Phylogenet. Evol 52, 735–745. doi: 10.1016/j.ympev.2009.05.017 19465138

[R55] Troussellier M, Escalas A, Bouvier T, and Mouillot D (2017). Sustaining rare marine microorganisms: macroorganisms as repositories and dispersal agents of microbial diversity. Front. Microbiol 8:947. doi: 10.3389/fmicb.2017.00947 28611749 PMC5447324

[R56] Tyberghein L, Verbruggen H, Pauly K, Troupin C, Mineur F, and De Clerck O (2012). Bio-ORACLE: a global environmental dataset for marine species distribution modelling. Global Ecol. Biogeogr. 21, 272–281. doi: 10.1111/j.1466-8238.2011.00656.x

[R57] Urbanczyk H, Ogura Y, Hendry TA, Gould AL, Kiwaki N, Atkinson JT, (2011). Genome sequence of *Photobacterium mandapamensis* strain svers. 1.1, the bioluminescent symbiont of the cardinal fish *Siphamia versicolor*. J. Bacteriol 193, 3144–3145. doi: 10.1128/JB.00370-11 21478348 PMC3133217

[R58] Wada M, Kamiya A, Uchiyama N, Yoshizawa S, Kita-Tsukamoto K, Ikejima K, (2006). LuxA gene of light organ symbionts of the bioluminescent fish *Acropoma japonicum* (Acropomatidae) and *Siphamia versicolor* (Apogonidae) forms a lineage closely related to that of *Photobacterium leiognathi ssp. mandapamensis*. FEMS Microbiol. Lett 260, 186–192. doi: 10.1111/j.1574-6968.2006.00322.x 16842343

[R59] Wägele H (2004). Potential key characters in Opisthobranchia (Gastropoda, Mollusca) enhancing adaptive radiation. Org. Divers. Evol 4, 175–188. doi: 10.1016/j.ode.2004.03.002

[R60] Wallin IE (1927). Symbionticism and the Origin of Species. Baltimore: Williams and Wilkins company.

[R61] Wolfe AJ, Millikan DS, Campbell JM, and Visick KL (2004). *Vibrio fischeri* sigma54 controls motility, biofilm formation, luminescence, and colonization. Appl. Environ. Microbiol 70, 2520–2524. doi: 10.1128/AEM.70.4.2520-2524.2004 15066853 PMC383144

[R62] Wollenberg MS, and Ruby EG (2009). Population structure of *Vibrio fischeri* within the light organs of Euprymna scolopes squid from two Oahu (Hawaii) populations. Appl. Environ. Microbiol 75, 193–202. doi: 10.1128/AEM.01792-08 18997024 PMC2612210

[R63] Zamborsky DJ, and Nishiguchi MK (2011). Phylogeographical patterns among mediterranean sepiolid squids and their vibrio symbionts: environment drives specificity among sympatric species. Appl. Environ. Microbiol 77, 642–649. doi: 10.1128/AEM.02105-10 21075896 PMC3020525

