# Supplementary material for: Museum Genomics Illuminate the High Specificity of a Bioluminescent Symbiosis for a Genus of Reef Fish
Source: Front Ecol Evol. Author manuscript; Available in PMC 2021 Sep 2. (PMC8412414; doi:10.3389/fevo.2021.630207)
Supplement: GouldEtal2021_SuppMat [file NIHMS1724650-supplement-GouldEtal2021_SuppMat.pdf]

**Supplementary Information**

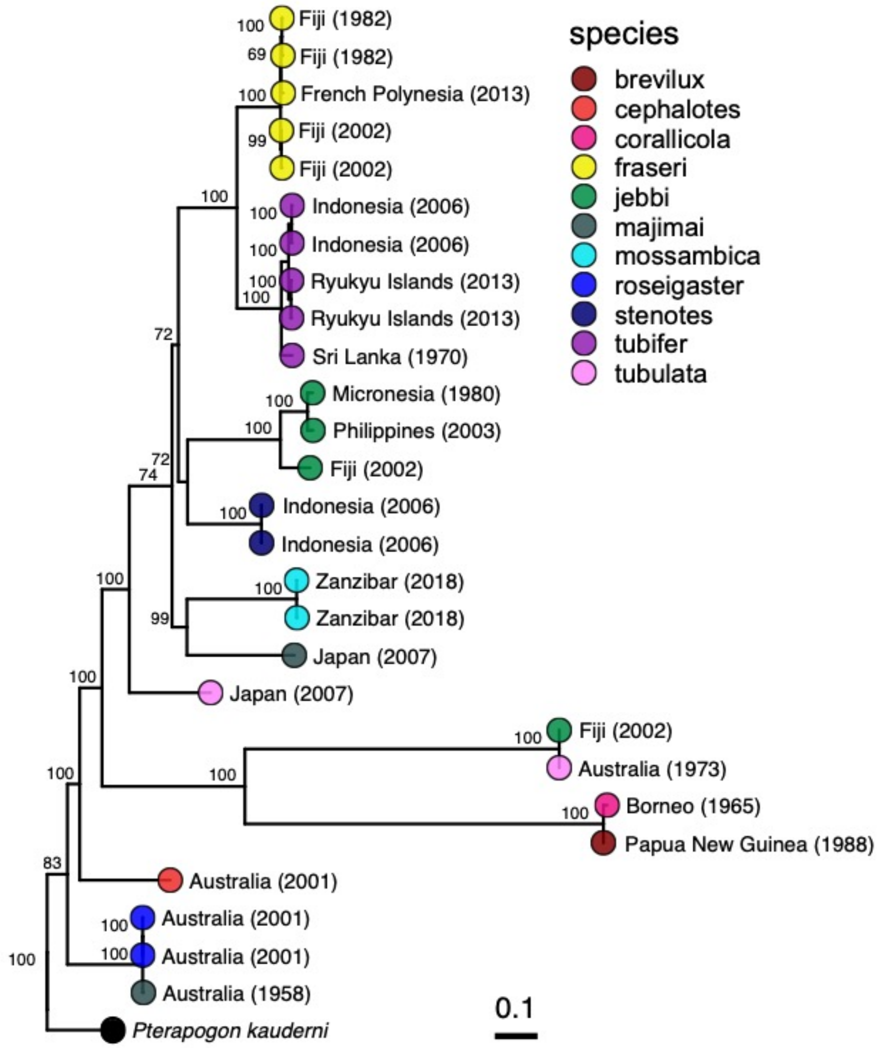


**Figure S1.** Maximum likelihood phylogeny of cardinalfishes in the genus *Siphamia* based on a concatenated supermatrix of 15 mtDNA gene sequences: *ATP6, ATP8, COXI, COX2, COX3, CYTB, ND1, ND2, ND3, ND4, ND4L, ND5, ND6, 16S, 18S*. Species identities are indicated by the branch tip colors and the sampling location and year of each specimen is listed in the branch label.

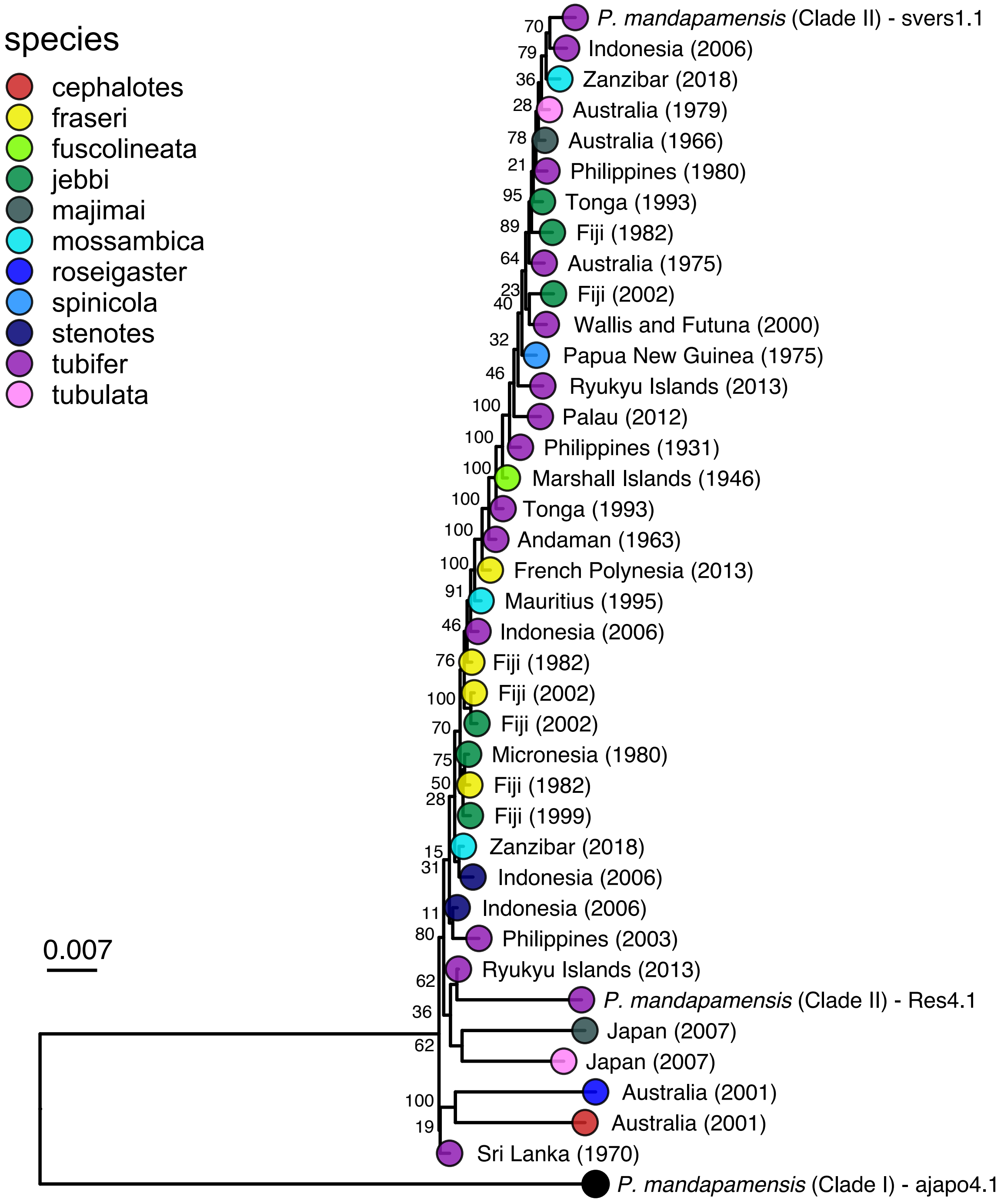


**Figure S2.** Maximum likelihood phylogeny of the light organ symbionts of various *Siphamia* species constructed from a core set of 165 single nucleotide polymorphisms. Corresponding host species are indicated by the branch tip colors and the sampling location and year of each specimen is listed in the branch label. Bootstrap support values are indicated at each node.

**Table S1**. Information for the *Siphamia* specimens sampled and their corresponding sequence information. Listed are each specimen's catalog number or unique identifier, species identification, sampling location and year, the standard length of the individual sampled, the total amount of double stranded DNA extracted from the light organ, the raw number of sequence reads, the number of reads that passed quality filtering and were trimmed, the number of reads that aligned to the symbiont reference genome (*P. mandapamensis* strain svers1.1), the percent of the symbiont reference genome covered at 10x sequence read depth, the total number of SNPs identified for each symbiont relative to the reference genome, the type of sequencing that was carried out, and the kit used for sequence library preparation. Specimens with decimals after their catalog number or unique identifier indicate that more than one individual was sampled from the specimen lot.

| Specimen ID | Species | Location | Year | Length (cm) | Total dsDNA (ng) | Raw | Trimmed | Aligned | %10x | SNPs | Sequence Run | Library Prep |
|---|---|---|---|---|---|---|---|---|---|---|---|---|
| AMI18353-041 | jebbi | Fiji | 1974 | 1.69 | <2 | 28258958 | 26861780 | 187425 | 0.5 | 0 | HiSeq 2x150 | Swift |
| | | | | | | 27257752 | 26799476 | 407326 | 1 | 166 | NovaSeq 2x150 | Swift |
| AMI18740-066 | jebbi | Australia | 1975 | 1.46 | <2 | 40906024 | 39209732 | 306641 | 2.1 | 0 | HiSeq 2x150 | Swift |
| AMI19450-018.1 | tubifer | Australia | 1975 | 2.94 | 3 | 58651761 | 56851489 | 442960 | 2.6 | 0 | HiSeq 2x150 | Swift |
| AMI19450-018.2 | tubifer | Australia | 1975 | 3.62 | 3.5 | 33895456 | 32610468 | 1337245 | 23.2 | 10 | HiSeq 2x150 | NEB Ultra II |
| | | | | | | 24526375 | 23858943 | 1042258 | 16.4 | 5,435 | NovaSeq 2x150 | NEB Ultra II |
| AMI20353-001 | majimai | Australia | 1972 | 1.67 | <2 | 39503975 | 38494612 | 344674 | 2.3 | 1 | HiSeq 2x150 | Swift |
| AMI20753-031 | tubulata | Australia | 1979 | 2.56 | <2 | 35965027 | 35217790 | 1085963 | 14.9 | 6,916 | NovaSeq 2x150 | Swift |
| AMI33715-016 | jebbi | Australia | 1993 | 1.46 | 2.2 | 35229540 | 33924547 | 359824 | 1.9 | 0 | HiSeq 2x150 | Swift |
| | | | | | | 33504802 | 33190608 | 655297 | 5 | 862 | NovaSeq 2x150 | Swift |
| AMI37933-007 | tubifer | Vanuatu | 1997 | 2.19 | 52.1 | 3604557 | 2090383 | 219645 | 0.1 | 47 | NovaSeq 2x150 | NEB Ultra II |
| | | | | | | 49624926 | 48314012 | 498902 | 1.9 | 0 | HiSeq 2x150 | Swift |
| AMI40838-008 | cephalotes | Australia | 2001 | 3.07 | 52.8 | 34771013 | 34092047 | 5099255 | 92.3 | 70,709 | NovaSeq 2x150 | NEB Ultra II |
| AMI40865-004.1 | roseigaster | Australia | 2001 | 4.56 | 74.1 | 91467705 | 89885499 | 6944692 | 93.4 | 72,219 | NovaSeq 2x150 | NEB Ultra II |
| AMI40865-004.2 | roseigaster | Australia | 2001 | 4.55 | 4.6 | 48426323 | 46531047 | 654416 | 5.9 | 1,588 | HiSeq 2x150 | Swift |
| AMIB4208 | majimai | Australia | 1958 | 2.31 | <2 | 36877472 | 34915450 | 467033 | 6.4 | 2 | HiSeq 2x150 | Swift |
| | | | | | | 87751 | 55995 | 12134 | 0 | 0 | NovaSeq 2x150 | Swift |

| | | | | | | | | | | | | |
|---|---|---|---|---|---|---|---|---|---|---|---|---|
| AMIB4247 | tubifer | Vanuatu | 1959 | 2.01 | <2 | 28358555 | 27422784 | 269641 | 2 | 0 | HiSeq 2x150 | Swift |
| | | | | | | 23520680 | 23130431 | 584903 | 2.9 | 1,633 | NovaSeq 2x150 | Swift |
| CAS247233.1 | mossambica | Zanzibar | 2018 | 2.55 | 542 | 5478617 | 5384608 | 454932 | 76.9 | 8,112 | HiSeq 1x150 | SparQ |
| CAS247233.2 | mossambica | Zanzibar | 2018 | 2.92 | 1530 | 75836474 | 73645413 | 33775734 | 96.4 | 15,579 | NovaSeq 2x150 | NEB Ultra II |
| CAS222309 | jebbi | Fiji | 2002 | - | 10.2 | 50665038 | 45699796 | 28523692 | 95.2 | 21,238 | NovaSeq 2x150 | NEB Ultra II |
| CAS223855 | jebbi | Fiji | 2002 | - | <2 | 44113541 | 41855479 | 377420 | 2.4 | 3 | HiSeq 2x150 | Swift |
| | | | | | | 33488272 | 33006205 | 576345 | 2.8 | 469 | NovaSeq 2x150 | Swift |
| CAS223939.1 | jebbi | Fiji | 2002 | 2.35 | 11.8 | 28012894 | 21498132 | 225577 | 4.3 | 63 | HiSeq 1x150 | SparQ |
| CAS223939.2 | jebbi | Fiji | 2002 | 1.81 | 8.9 | 11046726 | 10551137 | 716650 | 12 | 7,698 | NovaSeq 2x150 | NEB Ultra II |
| CAS223978.1 | unknown | Fiji | 2002 | 3.68 | 178.5 | 330421 | 323250 | 32364 | 0.1 | 10 | HiSeq 1x150 | SparQ |
| CAS223978.2 | unknown | Fiji | 2002 | 4.05 | 50.5 | 23063434 | 21244678 | 10747171 | 95.4 | 18,773 | NovaSeq 2x150 | NEB Ultra II |
| CAS223979.1 | fraseri | Fiji | 2002 | 2.8 | 9.2 | 24192691 | 22748353 | 328578 | 0.9 | 0 | HiSeq 2x150 | NEB Ultra II |
| | | | | | | 26735318 | 25635641 | 457797 | 1.1 | 7 | NovaSeq 2x150 | NEB Ultra II |
| CAS223979.2 | fraseri | Fiji | 2002 | 3.04 | 15.5 | 10467851 | 9981063 | 381078 | 17.7 | 72 | HiSeq 1x150 | SparQ |
| CAS225045 | jebbi | Fiji | 1999 | | 3.4 | 21590245 | 20304117 | 15184111 | 96.1 | 18,316 | NovaSeq 2x150 | NEB Ultra II |
| CAS27441 | tubifer | Philippines | 1931 | 3.26 | 1.8 | 18607665 | 18124484 | 1483134 | 95.3 | 18,841 | HiSeq 1x150 | SparQ |
| CAS28515 | tubulata | Australia | 1973 | - | <2 | 36559960 | 33708337 | 213670 | 1 | 2 | HiSeq 2x150 | Swift |
| | | | | | | 35922973 | 35323462 | 589696 | 3.1 | 1,301 | NovaSeq 2x150 | Swift |
| CAS84356 | tubifer | Palau | 2012 | 1.9 | 38.9 | 48287910 | 38767642 | 6258850 | 64.8 | 13,661 | HiSeq 1x150 | SparQ |
| Stubifer_M118 | tubifer | Ryukyu Islands | 2013 | 1.3 | 26.5 | 75237177 | 72661256 | 57068084 | 96.2 | 16,828 | HiSeq 2x150 | Swift |
| Smajimai_PVD | majimai | Japan | 2007 | 2.61 | 8949 | 36930530 | 35957911 | 26787137 | 94.8 | 70,889 | NovaSeq 2x150 | NEB Ultra II |
| Stubulata_PVD | tubulata | Japan | 2007 | 2.12 | 1225.5 | 34195315 | 33077310 | 21855337 | 95.1 | 66,583 | NovaSeq 2x150 | NEB Ultra II |
| Stubifer_S27 | tubifer | Ryukyu Islands | 2013 | 2.65 | - | 7403111 | 7277173 | 517331 | 88.9 | 19,790 | HiSeq 1x150 | SparQ |

| | | | | | | | | | | | | |
|---|---|---|---|---|---|---|---|---|---|---|---|---|
| Sstenotes_GRA.1 | stenotes | Indonesia | 2006 | 1.89 | 115.9 | 22315393 | 24640343 | 21426979 | 95.8 | 24,716 | NovaSeq 2x150 | NEB Ultra II |
| Sstenotes_GRA.2 | stenotes | Indonesia | 2006 | 1.98 | 308 | 467708 | 426870 | 85208 | 0.5 | 23 | HiSeq 1x150 | SparQ |
| Stubifer_GRA.1 | tubifer | Indonesia | 2006 | 2.39 | 96 | 23365446 | 22298866 | 6159687 | 95.8 | 18,701 | NovaSeq 2x150 | NEB Ultra II |
| Stubifer_GRA.2 | tubifer | Indonesia | 2006 | 2.85 | 95.9 | 9455319 | 8509771 | 452475 | 52 | 3,041 | HiSeq 1x150 | SparQ |
| USNM112099 | elongata | Philippines | 1909 | 3.46 | <2 | 49945895 | 48231023 | 430697 | 3.7 | 8 | HiSeq 2x150 | Swift |
| USNM142281.1 | fuscolineata | Marshall Islands | 1946 | 2.2 | <2 | 16213148 | 15893522 | 6118510 | 96.1 | 15,366 | NovaSeq 2x150 | Swift |
| USNM142281.2 | fuscolineata | Marshall Islands | 1946 | 2.76 | 10.1 | 39670892 | 27192174 | 695872 | 62.8 | 381 | HiSeq 1x150 | NEB Ultra II |
| USNM203781 | corallicola | Borneo | 1965 | 2.58 | <2 | 33588104 | 32488841 | 640895 | 0.6 | 0 | HiSeq 2x150 | Swift |
| | | | | | | 56446868 | 51658133 | 1237586 | 1.2 | 178 | NovaSeq 2x150 | Swift |
| USNM223216 | jebbi | Micronesia | 1980 | 1.74 | 7 | 181380283 | 186040521 | 176746273 | 96.5 | 14,693 | NovaSeq 2x150 | NEB Ultra II |
| USNM245638 | jebbi | Fiji | 1982 | 2.07 | 2.2 | 36426337 | 34932628 | 508273 | 9.2 | 38 | HiSeq 2x150 | Swift |
| | | | | | | 27073152 | 26801480 | 647893 | 13 | 2,188 | NovaSeq 2x150 | Swift |
| USNM245641 | fraseri | Fiji | 1982 | 4.13 | 5.3 | 864701674 | 829515210 | 714629012 | 97.5 | 17,070* | NovaSeq 2x150 | NEB Ultra II |
| USNM245642 | fraseri | Fiji | 1982 | 3.65 | 13 | 35743393 | 35774905 | 33716527 | 96.2 | 16,396 | NovaSeq 2x150 | NEB Ultra II |
| USNM298542 | brevilux | Papua New Guinea | 1988 | 2.24 | 21.1 | 662337 | 482006 | 148333 | 0.1 | 23 | NovaSeq 2x150 | NEB Ultra II |
| | | | | | | 49597285 | 47386221 | 486582 | 2.4 | 0 | HiSeq 2x150 | Swift |
| USNM341594 | jebbi | Tonga | 1993 | 1.91 | <2 | 27318206 | 27030306 | 1313295 | 61.4 | 7,995 | NovaSeq 2x150 | Swift |
| USNM341595 | tubifer | Tonga | 1993 | 3.87 | 7.8 | 18908174 | 17504661 | 4858215 | 94.2 | 725 | HiSeq 2x150 | NEB Ultra II |
| | | | | | | 11846770 | 11271717 | 2974122 | 88.1 | 17,900 | NovaSeq 2x150 | NEB Ultra II |
| USNM349778 | mossambica | Mauritius | 1995 | 2.36 | 15 | 33471029 | 32308812 | 12943283 | 95.8 | 18,219 | NovaSeq 2x150 | NEB Ultra II |
| USNM357884 | tubifer | Philippinnes | 1980 | 3.68 | 7.3 | 18374904 | 17191172 | 759889 | 8.5 | 1 | HiSeq 2x150 | NEB Ultra II |
| | | | | | | 13974418 | 12217495 | 879577 | 11.9 | 4,148 | NovaSeq 2x150 | NEB Ultra II |
| USNM357889 | spinicola | Papua New Guinea | 1975 | 3.11 | 4.1 | 21853900 | 21329666 | 2042137 | 42.7 | 12,366 | NovaSeq 2x150 | NEB Ultra II |

| | | | | | | | | | | | | |
|---|---|---|---|---|---|---|---|---|---|---|---|---|
| | | | | | | 46425 | 42876 | 22 | 0 | 0 | HiSeq 2x150 | NEB Ultra II |
| USNM357892 | tubifer | Red Sea | 1969 | 3.35 | <2 | 35509177 | 33451543 | 215301 | 0.7 | 1 | HiSeq 2x150 | Swift |
| | | | | | | 27788827 | 27434566 | 542781 | 1.7 | 1,107 | NovaSeq 2x150 | Swift |
| USNM357897 | tubifer | Andaman | 1963 | 4.09 | 3.9 | 26178035 | 25619280 | 9568200 | 95 | 17,295 | NovaSeq 2x150 | NEB Ultra II |
| USNM357999 | tubifer | Sri Lanka | 1970 | 2.94 | <2 | 54251311 | 53731832 | 26374339 | 95.6 | 22,417 | NovaSeq 2x150 | Swift |
| USNM358001 | majimai | Philippines | 1978 | 2.1 | <2 | 38324209 | 36313447 | 254445 | 0.8 | 2 | HiSeq 2x150 | Swift |
| | | | | | | 33631537 | 33293817 | 428773 | 1.1 | 151 | NovaSeq 2x150 | Swift |
| USNM374480 | majimai | Australia | 1966 | 1.97 | 2.1 | 63381524 | 62370257 | 1400945 | 40.5 | 8,157 | NovaSeq 2x150 | Swift |
| USNM374837 | unknown | Wallis and Futuna | 2000 | 1.96 | 12.1 | 10549216 | 7951371 | 985084 | 22.8 | 9,465 | NovaSeq 2x150 | NEB Ultra II |
| | | | | | | 41856128 | 40302628 | 595029 | 10.7 | 11 | HiSeq 2x150 | Swift |
| USNM396981 | stenotes | Indonesia | 2006 | 1.89 | 153.9 | 36161679 | 35712980 | 34096922 | 96.1 | 16,892 | NovaSeq 2x150 | NEB Ultra II |
| USNM412731 | jebbi | Philippines | 2003 | 1.73 | 23.6 | 27071135 | 25964385 | 16604538 | 95.9 | 32,687 | NovaSeq 2x150 | NEB Ultra II |
| USNM430718 | fraseri | French Polynesia | 2013 | 3.34 | 58.9 | 18468382 | 17513981 | 2806955 | 95.3 | 20,592 | NovaSeq 2x150 | NEB Ultra II |

**Table S2**. Information for the *Siphamia COI* sequences that were used to construct the host phylogeny. Listed are each specimen's catalog number or unique identifier, species identification, sampling location, exact latitude and longitude, year, and the source of the sequence.

| Specimen ID | Species | Location | Latitude | Longitude | Year | Source |
|---|---|---|---|---|---|---|
| AMI40838-008 | cephalotes | Australia | -33.84 | 151.19 | 2001 | this study |
| AMI40865-004-1 | roseigaster | Australia | -33.87 | 152.00 | 2001 | this study |
| AMI40865-004-2 | roseigaster | Australia | -33.87 | 152.00 | 2001 | this study |
| AMI41858-030 | roseigaster | Australia | -29.42 | 153.36 | 2002 | Mabuchi *et al.* 2014 |
| AWCF412 | goreni | Red Sea | 25.71 | 36.62 | 2016 | Atta *et al*. 2019 |
| AWCF713 | tubifer | Red Sea | 25.71 | 36.62 | 2016 | Atta *et al*. 2019 |
| BW-A5255 | fistulosa | Australia | -16.90 | 146.45 | 2005 | International Barcode of Life |
| CAS223855 | jebbi | Fiji | -18.15 | 178.36 | 2002 | this study |
| CAS223978 | fraseri | Fiji | -18.10 | 178.36 | 2002 | this study |
| CAS223979 | fraseri | Fiji | -18.10 | 178.36 | 2002 | this study |
| CAS225045 | jebbi | Fiji | -18.15 | 178.37 | 1999 | this study |
| CAS247233.1 | mossambica | Zanzibar | -6.22 | 39.17 | 2018 | this study |
| CAS247233.2 | mossambica | Zanzibar | -6.22 | 39.17 | 2018 | this study |
| CAS28515 | tubulata | Australia | -14.20 | 144.26 | 1973 | this study |
| CSIRO-H-6648-02 (BW-A12333) | guttulata | Australia | -17.11 | 146.01 | 2004 | International Barcode of Life |
| CSIRO-H-7457-03 (BW-A12338) | guttulata | Australia | -12.58 | 143.48 | 2004 | International Barcode of Life |
| CSIRO-H-8482-02 (BW-A5590) | cuneiceps | Australia | -22.12 | 150.33 | 2005 | International Barcode of Life |
| FAKU73087 | tubulata | Japan | 32.74 | 132.56 | - | Mabuchi *et al.* 2014 |
| FAKU78690 | majimai | Ryukyu Islands | 30.43 | 130.40 | - | Mabuchi *et al.* 2014 |
| KU_Tissue4631 (CAS222309) | jebbi | Fiji | -17.32 | 178.24 | 2002 | Mabuchi *et al.* 2014 |
| Stubifer_M118 | tubifer | Ryukyu Islands | 26.66 | 127.88 | 2013 | this study |
| Smajimai_PVD | majimai | Japan | 32.80 | 133.50 | 2007 | this study |
| Stubulata_PVD | tubulata | Japan | 32.74 | 132.56 | 2007 | this study |
| Stubifer_S27 | tubifer | Ryukyu Islands | 26.64 | 127.87 | 2013 | this study |
| SAIAB194663 | paupuensis | Indonesia | -2.22 | 130.56 | 2013 | Gon *et al*. 2014 |

| | | | | | | |
|---|---|---|---|---|---|---|
| SAIAB194704 | paupuensis | Indonesia | -2.97 | 131.33 | 2013 | Gon *et al*. 2014 |
| Sstenotes_GRA.1 | stenotes | Indonesia | -3.87 | 133.98 | 2006 | this study |
| Sstenotes_GRA.2 | stenotes | Indonesia | -3.87 | 133.98 | 2006 | this study |
| Stubif_Kaeding | tubifer | Ryukyu Islands | 26.64 | 127.87 | 2006 | Kaeding *et al.* 2007 |
| Stubifer_GRA.1 | tubifer | Indonesia | -3.68 | 133.73 | 2006 | this study |
| Stubifer_GRA.2 | tubifer | Indonesia | -3.68 | 133.73 | 2006 | this study |
| USNM112099-2 | elongata | Philippines | 16.93 | 120.23 | 1909 | this study |
| USNM203781 | corallicola | Borneo | 6.02 | 116.06 | 1965 | this study |
| USNM223216 | jebbi | Micronesia | 6.93 | 158.10 | 1980 | this study |
| USNM245638 | jebbi | Fiji | -19.16 | 179.76 | 1982 | this study |
| USNM245641 | fraseri | Fiji | -20.62 | 181.33 | 1982 | this study |
| USNM245642 | fraseri | Fiji | -20.62 | 181.33 | 1982 | this study |
| USNM298542-2 | brevilux | Papua New Guinea | -5.23 | 145.75 | 1988 | this study |
| USNM349778 | mossambica | Mauritius | -20.19 | 57.40 | 1995 | this study |
| USNM357999 | tubifer | Sri Lanka | 8.60 | 81.23 | 1970 | this study |
| USNM358001 | majimai | Philippines | 9.38 | 123.26 | 1978 | this study |
| USNM396981 | stenotes | Indonesia | -3.96 | 134.36 | 2006 | this study |
| USNM412731 | jebbi | Philippines | 12.69 | 120.52 | 2003 | this study |
| USNM430718 | fraseri | French Polynesia | -22.64 | 207.18 | 2013 | this study |

Atta, C. J., Coker, D. J., Sinclair-Taylor, T. H., DiBattista, J. D., Kattan, A., Monroe, A. A., & Berumen, M. L. (2019). Conspicuous and cryptic reef fishes from a unique and economically important region in the northern Red Sea. *PloS one*, *14*(10), e0223365.

Gon, O., and Allen, G. R. (2012). Revision of the Indo-Pacific cardinalfish genus Siphamia (Perciformes: Apogonidae). *Zootaxa* 3294, 1–84. doi: 10.11646/zootaxa.3294.1.1

Kaeding, A. J., Ast, J. C., Pearce, M. M., Urbanczyk, H., Kimura, S., Endo, H., et al. (2007). Phylogenetic diversity and cosymbiosis in the bioluminescent symbioses of “Photobacterium mandapamensis”. *Appl. Environ. Microbiol.* 73, 3173–3182. doi: 10.1128/AEM.02212-06

Mabuchi, K., Fraser, T. H., Song, H., Azuma, Y., and Nishida, M. (2014). Revision of the systematics of the cardinalfishes (Percomorpha: Apogonidae) based on moecular analyses and comparative reevaluation of morphological characters. *Zootaxa* 3846, 151–203 doi: 10.11646/zootaxa.3846.2.1

**Table S3**. Results of the nucleotide BLAST search of symbiont 16S rRNA genes. Listed are each specimen's catalog number or unique identifier, the percent of the reference 16S rRNA gene sequence (*Photobacterium leiognathi*, AY292917) covered at 10x sequence depth, the top matching sequence from the NCBI database including its accession number in parentheses, and the corresponding query coverage, E-value, and percent identity relative to that sequence.

| Specimen ID | %10x | Top hit | Query coverage | E-value | % identity |
|---|---|---|---|---|---|
| AMI18353-041 | 88.49 | Photobacterium leiognathi subsp. mandapamensis strain MahLm3 (JN380344.1) | 91% | 0.0 | 86.08 |
| AMI18740-066 | 89.2 | Photobacterium leiognathi (AY292917.1) | 93% | 0.0 | 79.97 |
| AMI19450-018.1 | 98.25 | Photobacterium leiognathi strain LC1-277 (AB243248.1) | 90% | 0.0 | 86.48 |
| AMI19450-018.2 | 99.94 | Photobacterium leiognathi strain ljone1.1 (AY204494.1) | 94% | 0.0 | 95.96 |
| AMI20353-001 | 95.02 | Photobacterium leiognathi strain W214 (MF554624.1) | 89% | 0.0 | 83.82 |
| AMI20753-031 | 99.94 | Photobacterium leiognathi strain ljone1.1 (AY204494.1) | 94% | 0.0 | 95.14 |
| AMI33715-016 | 100 | Photobacterium mandapamensis seafl.1.4 (AY455873.1) | 95% | 0.0 | 84.58 |
| AMI37933-007 | 99.81 | Photobacterium mandapamensis seafl.1.4 (AY455873.1) | 95% | 0.0 | 95.93 |
| AMI40838-008 | 100 | Photobacterium leiognathi subsp. mandapamensis strain ATCC 27561 (NR_115206.1) | 95% | 0.0 | 99.93 |
| AMI40865-004.1 | 99.94 | Photobacterium leiognathi subsp. mandapamensis strain ATCC 27561 (NR_115206.1) | 95% | 0.0 | 99.86 |
| AMI40865-004.2 | 99.94 | Photobacterium leiognathi strain AK-MIE (MH746214.1) | 91% | 0.0 | 99.01 |
| AMIB4208 | 100 | Photobacterium leiognathi strain LC1-283 (AB243249.1) | 90% | 0.0 | 90.74 |
| AMIB4247 | 99.94 | Photobacterium leiognathi strain LC1-283 (AB243249.1) | 90% | 0.0 | 92.75 |
| CAS222309 | 100 | Photobacterium leiognathi subsp. mandapamensis strain ATCC 27561 (NR_115206.1) | 95% | 0.0 | 100.00 |
| CAS223855 | 100 | Photobacterium leiognathi strain LC1-283 (AB243249.1) | 88% | 0.0 | 86.35 |
| CAS223939.1 | 99.94 | Photobacterium leiognathi strain ljone1.1 (AY204494.1) | 94% | 0.0 | 99.04 |
| CAS223939.2 | 99.94 | Photobacterium mandapamensis seafl.1.1 (AY455871.1) | 94% | 0.0 | 97.67 |
| CAS223978.1 | 29.61 | Photobacterium leiognathi subsp. mandapamensis strain ATCC 27561 (NR_115206.1) | 95% | 0.0 | 99.73 |
| CAS223978.2 | 100 | Photobacterium mandapamensis seafl.1.1 (AY455871.1) | 94% | 0.0 | 100.00 |
| CAS223979.1 | 99.94 | Photobacterium leiognathi strain LC1-277 (AB243248.1) | 90% | 0.0 | 94.88 |

| | | | | | |
|---|---|---|---|---|---|
| CAS223979.2 | 99.94 | Photobacterium leiognathi subsp. mandapamensis strain ATCC 27561 (NR_115206.1) | 95% | 0.0 | 100.00 |
| CAS225045 | 100 | Photobacterium leiognathi subsp. mandapamensis strain ATCC 27561 (NR_115206.1) | 95% | 0.0 | 100.00 |
| CAS247233.1 | 100 | Photobacterium mandapamensis seafl.1.1 (AY455871.1) | 94% | 0.0 | 100.00 |
| CAS247233.2 | 99.94 | Photobacterium leiognathi subsp. mandapamensis strain ATCC 27561 (NR_115206.1) | 95% | 0.0 | 100.00 |
| CAS27441 | 100 | Photobacterium leiognathi subsp. mandapamensis strain ATCC 27561 (NR_115206.1) | 95% | 0.0 | 100.00 |
| CAS28515 | 100 | Photobacterium leiognathi subsp. mandapamensis strain MahLm3 (JN380344.1) | 92% | 0.0 | 84.13 |
| CAS84356 | 99.94 | Photobacterium leiognathi subsp. mandapamensis strain ATCC 27561 (NR_115206.1) | 95% | 0.0 | 99.93 |
| Smajimai_PVD | 100 | Photobacterium leiognathi subsp. mandapamensis strain ATCC 27561 (NR_115206.1) | 95% | 0.0 | 99.93 |
| Sstenotes_GRA.1 | 100 | Photobacterium leiognathi subsp. mandapamensis strain ATCC 27561 (NR_115206.1) | 95% | 0.0 | 100.00 |
| Sstenotes_GRA.2 | 99.94 | Photobacterium leiognathi subsp. mandapamensis strain ATCC 27561 (NR_115206.1) | 95% | 0.0 | 99.73 |
| Stubifer_GRA.1 | 100 | Photobacterium leiognathi subsp. mandapamensis strain ATCC 27561 (NR_115206.1) | 95% | 0.0 | 100.00 |
| Stubifer_GRA.2 | 99.94 | Photobacterium leiognathi subsp. mandapamensis strain ATCC 27561 (NR_115206.1) | 95% | 0.0 | 99.93 |
| Stubifer_M118 | 100 | Photobacterium leiognathi subsp. mandapamensis strain ATCC 27561 (NR_115206.1) | 95% | 0.0 | 99.93 |
| Stubifer_S27 | 99.94 | Photobacterium leiognathi subsp. mandapamensis strain ATCC 27561 (NR_115206.1) | 95% | 0.0 | 100.00 |
| Stubulata_PVD | 100 | Photobacterium leiognathi subsp. mandapamensis strain ATCC 27561 (NR_115206.1) | 95% | 0.0 | 99.93 |
| USNM112099 | 92.18 | Photobacterium leiognathi strain AK5 (AB243232.1) | 90% | 0.0 | 85.57 |
| USNM142281.1 | 99.94 | Photobacterium leiognathi subsp. mandapamensis strain ATCC 27561 (NR_115206.1) | 95% | 0.0 | 100.00 |
| USNM142281.2 | 100 | Photobacterium leiognathi strain ljone1.1 (AY204494.1) | 93% | 0.0 | 94.36 |
| USNM203781 | 100 | Photobacterium leiognathi strain LC1-277 (AB243248.1) | 90% | 0.0 | 90.68 |
| USNM223216 | 100 | Photobacterium leiognathi subsp. mandapamensis strain ATCC 27561 (NR_115206.1) | 95% | 0.0 | 100.00 |
| USNM245638 | 100 | Photobacterium leiognathi strain AK5 (AB243232.1) | 90% | 0.0 | 93.11 |
| USNM245641 | 100 | Photobacterium leiognathi subsp. mandapamensis strain ATCC 27561 (NR_115206.1) | 95% | 0.0 | 99.93 |
| USNM245642 | 100 | Photobacterium leiognathi subsp. mandapamensis strain ATCC 27561 (NR_115206.1) | 95% | 0.0 | 100.00 |

| | | | | | |
|---|---|---|---|---|---|
| USNM298542 | 99.94 | Photobacterium leiognathi subsp. mandapamensis strain MahLm3 (JN380344.1) | 91% | 0.0 | 82.60 |
| USNM341594 | 100 | Photobacterium leiognathi subsp. mandapamensis strain MahLm3 (JN380344.1) | 92% | 0.0 | 93.40 |
| USNM341595 | 100 | Photobacterium leiognathi subsp. mandapamensis strain ATCC 27561 (NR_115206.1) | 95% | 0.0 | 100.00 |
| USNM349778 | 100 | Photobacterium leiognathi subsp. mandapamensis strain ATCC 27561 (NR_115206.1) | 95% | 0.0 | 99.93 |
| USNM357884 | 100 | Photobacterium mandapamensis seafl.1.1 (AY455871.1) | 94% | 0.0 | 100.00 |
| USNM357889 | 100 | Photobacterium mandapamensis seafl.1.4 (AY455873.1) | 95% | 0.0 | 97.90 |
| USNM357892 | 100 | Photobacterium leiognathi strain LC1-277 (AB243248.1) | 90% | 0.0 | 91.53 |
| USNM357897 | 100 | Photobacterium leiognathi subsp. mandapamensis strain ATCC 27561 (NR_115206.1) | 95% | 0.0 | 99.93 |
| USNM357999 | 100 | Photobacterium leiognathi subsp. mandapamensis strain ATCC 27561 (NR_115206.1) | 95% | 0.0 | 99.93 |
| USNM358001 | 100 | Photobacterium leiognathi strain LC1-277 (AB243248.1) | 90% | 0.0 | 87.62 |
| USNM374480 | 100 | Photobacterium leiognathi strain LC1-283 (AB243249.1) | 90% | 0.0 | 91.82 |
| USNM374837 | 100 | Photobacterium leiognathi subsp. mandapamensis strain MahLm3 (JN380344.1) | 91% | 0.0 | 87.07 |
| USNM396981 | 100 | Photobacterium leiognathi strain lleuc1.1 (AY204495.1) | 94% | 0.0 | 100.00 |
| USNM412731 | 100 | Photobacterium leiognathi subsp. mandapamensis strain ATCC 27561 (NR_115206.1) | 95% | 0.0 | 100.00 |
| USNM430718 | 99.94 | Photobacterium leiognathi subsp. mandapamensis strain ATCC 27561 (NR_115206.1) | 95% | 0.0 | 100.00 |
